# Globo H ceramide confers chemoresistance and poor prognosis to advanced gallbladder cancer via A2AR/cAMP/PKA pathway

**DOI:** 10.7150/thno.126092

**Published:** 2026-02-26

**Authors:** Tsai-Hsien Hung, Jung-Tung Hung, Wen-Kuan Huang, Yenlin Huang, Chiao-En Wu, Jing-Yan Cheng, Chien-Wei Lee, John Yu, Yi-Chia Wu, Chun-Nan Yeh, Alice L. Yu

**Affiliations:** 1 Institute of Stem Cell and Translational Cancer Research, Chang Gung Memorial Hospital at Linkou, Taoyuan, Taiwan.; 2 Department of Hematology-Oncology, Chang Gung Memorial Hospital, Chang Gung University, Taoyuan, Taiwan.; 3 Department of Anatomic Pathology, Chang Gung Memorial Hospital at Linkou, Taoyuan, Taiwan.; 4 School of medicine, National Tsing Hua University, Hsinchu, Taiwan.; 5 Chang Gung University, Taoyuan, Taiwan.; 6 Division of Breast Oncology and Surgery, Department of Surgery, Kaohsiung Medical University Hospital, Kaohsiung Medical University, Kaohsiung, Taiwan.; 7 Department of Surgery and Liver Research Center, Chang Gung Memorial Hospital, Chang Gung University, Taoyuan, Taiwan.; 8 Department of Pediatrics, University of California in San Diego, San Diego, CA 92103, USA.

**Keywords:** Globo H, gallbladder cancer, gemcitabine, immunotherapy, prognosis

## Abstract

**Rationale:**

Gallbladder cancer (GBC) has poor prognosis, is primarily treated with gemcitabine-based chemotherapy, which is limited by gemcitabine correlates with GBC progression. This study investigate the role of Globo H ceramide (GHCer) in GR and explore whether targeting Glob H could overcome resistance**.**

**Methods:**

Globo H expression was assessed by immunohistochemistry in 81 GBC samples. GHCer-induced GR and ABCG2 upregulation were assessed in GBC cell lines, patient-derived xenograft (PDX), and a thioacetamide (TAA)-induced rat cholangiocarcinoma model. A2AR/cAMP/PKA signaling involvement was examined using inhibitors and siRNA. The efficacy of anti-Globo H antibody (mAb VK9) or vaccine (OBI-833/OBI-821) combined with gemcitabine was evaluated *in vitro* and *in vivo*. Immune responses were assessed by ELISA and multiplex immunohistochemistry.

**Results:**

High Globo H expression correlated with shorter survival in GBC patients receiving gemcitabine. GHCer promoted GR via A2AR/cAMP/PKA-mediated ABCG2 upregulation, which was reversed by mAb VK9 or pathway inhibition. mAb VK9 or OBI-833/821 enhanced gemcitabine efficacy in GBC PDX and TAA cholangiocarcinoma models. OBI-833/821 induced anti-Globo H IgG/IgM, reduced Foxp3⁺ Tregs, and increased CD161⁺ NK cells in TAA model. A compassionate clinical use of 833/821 led to stable disease.

**Conclusions:**

GHCer promotes GR by upregulating ABCG2 via A2AR/cAMP/PKA signaling. Targeting Globo H may improve chemotherapy response in GBC.

## Introduction

Gallbladder cancer (GBC) is an aggressive malignancy that poses a significant healthcare challenge worldwide. It is often diagnosed at an advanced, unresectable stage, making systemic therapy as the primary treatment option 1, 2. Traditionally, GBC has been grouped with other biliary tract cancers in clinical trials, leading to the adoption of similar chemotherapy protocols for GBC and cholangiocarcinoma (CCA). These typically include gemcitabine and cisplatin as first-line therapy and FOLFOX as second-line therapy 3. However, recent biomarker-driven trials are beginning to reshape the cancer treatment paradigms for GBC and CCA. Advances in molecular profiling have identified genetic differences that could lead to more targeted therapeutic approaches 4, 5. Common genetic alterations, including Her2/neu amplification, PIK3CA mutations, FGFR fusions/rearrangements, BRAF mutations, and DNA repair genetic aberrations, represent actionable targets for treatment 6. These molecular markers offer new strategies to enhance the efficacy of standard chemotherapy and potentially improve the outcomes of patients with GBC and CCA. However, the complex etiology, diverse genetic alterations, and regional variations in tumor characteristics contribute to significant heterogeneity, complicating the translation of molecular findings into clinical practice. Clinical trials aimed at exploring these novel therapeutics in chemoresistant and recurrent GBC cases have yielded mixed results, highlighting the need for further research and personalized treatment strategies 6.

Gemcitabine resistance (GR) in advanced GBC is a complex and multifactorial phenomenon, driven by various genetic and molecular mechanisms 7. Researchers have identified several genes and pathways that contribute to GR, including hENT1, dCK, CNT3, BRCA1, BRCA2, RAD51, RRM1, RRM2, BCL-2, BCL-XL, and ATP-binding cassette (ABC) transporters 7-10. It is crucial to note that GR often results from a combination of genetic and epigenetic modifications within cancer cells, making it a particularly challenging issue to address. Moreover, the specific genes and pathways involved can vary among individuals and cancer subtypes, highlighting the complexity of the resistance mechanisms. A deeper understanding of these genetic and epigenetic factors is crucial for guiding personalized treatment strategies and developing more effective targeted therapies for advanced GBC.

The approval of dinutuximab, an anti-GD2 (disialoganglioside) antibody, for treating neuroblastoma has sparked interest in targeting tumor-associated carbohydrate antigens 11, 12. Glycomic profiling of tumor tissues has revealed consistent alterations in the N- and O-glycosylation patterns of surface glycoproteins and the aberrant expression of glycosphingolipids 13. These changes contribute to cancer proliferation, invasion, angiogenesis, and metastasis 13. Similar alterations have been observed in CCA cell lines, which exhibit aberrant expression of high-mannose glycans, sWGA-specific glycans, SJA-specific N-acetylgalactosamine-associated glycans, and elevated levels of hexosylceramides and lactosylceramides 14-16. These findings suggest that sWGA-specific glycans may be relevant biomarkers for bile duct cancer. However, glycosphingolipids have not been examined in these studies. Recent evidence suggests that GR in GBC may also be driven by ABC transporters, particularly ATP-binding cassette sub-family G member 2 (ABCG2), whose expression is regulated by the cyclic AMP/protein kinase A (cAMP/PKA) pathway 17-19. Our prior studies demonstrated that Globo H ceramide (GHCer) activates cAMP/PKA signaling via adenosine A2A receptor (A2AR) engagement 20, raising the possibility that GHCer may promote GR by upregulating ABCG2 through the A2AR/cAMP/PKA axis.

Asides from high-mannose glycans mentioned above, GHCer, a glycosphingolipid, has been shown to be widely overexpressed on the surface of various epithelial cancers, including those of the breast, colon, lung, stomach, prostate, pancreas, and ovary 21-24. Our previous research has demonstrated that GHCer plays a crucial role in promoting tumor angiogenesis and immune evasion, providing a strong rationale for developing Globo H-targeted immunotherapies 25-27. Notably, encouraging results from a multinational phase 2 randomized controlled trial of a Globo H vaccine in metastatic breast cancer revealed a low toxicity profile and significant survival benefits for patients generating effective humoral immune responses against Globo H 28.

While our earlier studies identified Globo H as a prognostic factor in intrahepatic CCA (iCCA) and resectable GBC 29, 30, its role in advanced unresectable GBC remains underexplored. In the present study, we showed that Globo H is expressed in advanced GBC and correlates with poorer progression-free and overall survival in patients undergoing gemcitabine treatment.

While GHCer is recognized as the most prevalent tumor-associated glycan, and its overexpression has been correlated with poor prognosis in various cancers, its role in drug resistance has not been previously reported. In this study, we identified GHCer as a critical mediator of GR in GBC through upregulation of ABCG2.Additionally, we demonstrated that GHCer positively regulates ABCG2 expression through A2AR and its downstream cAMP/PKA signaling, thereby contributing to GR. Furthermore, we provided preclinical evidence supporting the efficacy of Globo H-targeted therapy for GBC in combination with gemcitabine, using a GBC patient-derived xenograft (PDX) model and a thioacetamide (TAA)-induced rat CCA model. These findings were further validated by the clinical findings of a patient with advanced GBC who had disease progression on chemotherapy prior to receiving the Globo H vaccine. Collectively, our findings suggest a promising new direction for the treatment of advanced GBC by targeting GHCer.

## Materials and Methods

### Cell Lines

The following GBC cell lines were used in this study: SNU308 (human gallbladder adenocarcinoma cell line), TGBC24 (human gallbladder carcinoma cell line), and GBC01, a patient-derived primary cell line cultured from a GBC tumor specimen. SNU308 and GBC01 were cultured in RPMI-1640 medium (Corning, NY, USA) supplemented with 10% fetal bovine serum (FBS, Gibco, Thermo Fisher Scientific, MA, USA) and 1% penicillin-streptomycin (Gibco, Thermo Fisher Scientific, MA, USA), while TGBC24 was cultured in Dulbecco's Modified Eagle Medium (DMEM, Corning, NY, USA) with the same supplements. All cells were maintained at 37 °C in a humidified atmosphere containing 5% CO_2_. Cells were passaged every 2-3 days to maintain logarithmic growth and were regularly tested for mycoplasma contamination. GBC01 is a patient-derived primary cell line established from a gallbladder tumor specimen resected at Linkou Chang Gung Memorial Hospital with written informed consent and Institutional Review Board (IRB) approval (IRB No. 201901646B0A3).

### Clinical Samples and Compassionate-Use Case

Formalin-fixed paraffin-embedded (FFPE) tumor samples from 81 patients with unresectable GBC who received gemcitabine-based chemotherapy between 1990 and 2022 were obtained from the Linkou Chang Gung Memorial Hospital (CGMH) biobank (Taoyuan, Taiwan). Written informed consent was obtained from all patients, and study protocols were approved by the Institutional Review Board of CGMH (IRB No. 202201870B0). In addition, a patient with advanced, recurrent GBC who had progressed on gemcitabine-based chemotherapy was treated under a compassionate-use program with the OBI-833/OBI-821, a therapeutic vaccine comprising Globo H conjugated to CRM197 and co-administered with a saponin-based adjuvant. Use of the vaccine was approved by the Taiwan Food and Drug Administration (TFDA No. 1109031887). The patient received four weekly doses followed by biweekly and monthly booster injections. Serum levels of anti-Globo H IgG and IgM antibodies were monitored by ELISA at defined time points (weeks 0, 2, 6, 8, 12, and 16). Tumor response was assessed through imaging studies and tumor marker trends.

### Drug-response and Cell-viability Assay

For drug-response and cell-viability assays, SNU308, TGBC24, and GBC01 cells (5 × 10³/well) were seeded in 96-well plates and treated for 72 h with gemcitabine (0.1 nM-10 μM) in the presence or absence of GHCer (Tokyo Chemical Industry Co., Tokyo, Japan), glucosylceramide (GlcCer; Sigma-Aldrich, Cat# 131304P), anti-Globo H monoclonal antibody (mAb VK9, 10 μg/mL), or pathway inhibitors (H89, MedChemExpress, NJ, USA, 10 μM; ZM241385, Sigma-Aldrich, MO, USA, 5 μM). GlcCer was used as a structural control because it shares the same ceramide backbone as GHCer but lacks the terminal Globo H glycan, allowing us to differentiate the biological effects specifically attributable to the tumor-associated glycan moiety. H89 and ZM241385 were dissolved in DMSO and applied 1 h prior to GHCer treatment. Cell viability was assessed using the alamarBlue assay (Thermo Fisher Scientific, MA, USA). After 72 h of incubation, 10 μL of alamarBlue reagent was added to each well, followed by incubation for 4 h at 37°C. Fluorescence was measured at 570 nm excitation and 600 nm emission using a plate reader. IC_50_ values were calculated using GraphPad Prism 6.0 software. (GraphPad Software, La Jolla, CA, USA).

### cAMP Assays

According to the manufacturer's instructions, cAMP levels were measured using the LANCE cAMP assay kit (PerkinElmer, Waltham, MA, USA). SNU308 and TGBC24 cells (1 × 10^6^) were incubated with 30 μM GHCer. At the indicated time points, supernatants were collected to determine cAMP levels.

### siRNA Knock-down and cAMP Measurement

For siRNA experiments, SNU308, TGBC24, and GBC01 cells were transfected with small interfering RNAs (siRNAs) targeting PRKACA (sc-36240, Santa Cruz Biotechnology, USA), A2AR (sc-39850, Santa Cruz Biotechnology, USA) or ABCG2 (sc-41151, Santa Cruz Biotechnology, USA) using Lipofectamine RNAiMAX (Thermo Fisher Scientific, MA, USA) according to the manufacturer's instructions. Briefly, cells were seeded into 6-well plates at a density of 1 × 10^5^ cells/well, and transfection complexes were prepared by mixing siRNAs with Lipofectamine RNAiMAX in Opti-MEM medium (Thermo Fisher Scientific, MA, USA). After 48 h of transfection, cells were harvested for protein analysis and cell viability assays.

### TAA-Induced Rat CCA Model

To establish the CCA model, male Sprague-Dawley rats (6-8 weeks old) were treated with TAA via intraperitoneal injection (200 mg/L) in drinking water for 6 months to induce bile duct tumors 29. After tumor development, the rats were randomly divided into four groups (n = 8 per group): (1) control (PBS), (2) gemcitabine alone (Gem, 25 mg/kg), (3) OBI833 (30 µg OBI 833 + 100 µg OBI 821, SQ), and (4) combination therapy (Gem + OBI833). Treatments were administered biweekly for 2 months. Tumor metabolic activity was monitored using Positron emission tomography (PET) imaging at baseline, 1 month, and 2 months. At the end of the study, rats were sacrificed, and tumors were harvested for histological analysis and immunohistochemistry (IHC). All animal procedures were approved by the Experimental Animal Ethics Committee of Chang Gung Memorial Hospital (license number: 2022092605). This study followed the ARRIVE guidelines^31^.

### Enzyme-linked Immunosorbent Assay (ELISA) for the Determination of Anti-Globo H Titers in TAA Rat Model

Serum samples were collected from rats in the TAA-induced CCA rat model at three time points: pre-immune, the first PET scan, and the second PET scan. Rats were treated with OBI-833 + OBI-821 (Globo H vaccine), and anti-Globo H titers were measured using ELISA, following a previously described protocol 28. Optical density (OD) values obtained from GH-coated plates were corrected by subtracting the OD values from uncoated plates. For detection, Goat Anti-Rat IgG H&L horseradish peroxidase (HRP) (ab97057, Abcam) and Goat Anti-Rat IgM mu chain (HRP) (ab97180, Abcam) were used as secondary antibodies to assess IgG and IgM responses, respectively. The cut-off value was determined by adding 0.1 to the mean OD value of the negative control (secondary antibody only). The highest serum dilution with an absorbance greater than or equal to the cut-off value was recorded as the antibody titer. If the absorbance of the sample at a 20-fold dilution was below the cut-off value, the titer was recorded as 0.

### Multiplex Immunohistochemical Staining

Multiplex immunohistochemical analysis was performed on rat CCA tumor specimens to enable simultaneous detection and quantitative assessment of tumor cells and immune cell populations within the tumor microenvironment. The following markers were evaluated: Pan-cytokeratin (PanCK) as a tumor epithelial marker, Globo H as a tumor-associated glycosphingolipid, Foxp3 as a regulatory T cell marker, and CD161 as a natural killer (NK) cell marker. FFPE tissue sections were first incubated at 60 °C for 30 min, followed by deparaffinization using Dewax solution (Leica Biosystems, Buffalo Grove, IL, USA). Antigen retrieval was performed with ER2 retrieval solution (Leica Biosystems, IL, USA) at 100 °C for 30 min. After cooling, slides were washed and treated with 3% hydrogen peroxide to quench endogenous peroxidase activity. Sequential rounds of staining were performed using the Opal 7-color multiplex IHC system (Akoya Biosystems, Marlborough, MA, USA). For each staining cycle, slides were incubated with a primary antibody, followed by Opal polymer HRP Ms + Rb secondary antibody (Akoya Biosystems). Tyramide signal amplification (TSA) fluorophores were then applied, after which heat-induced stripping was performed to remove bound primary and secondary antibodies before the next staining cycle. Two multiplex staining panels were used. In both panels, Globo H was detected using the anti-Globo H monoclonal antibody VK9 (2.5 μg/mL, 20 min incubation) and visualized with Opal 520. PanCK was detected using an anti-PanCK antibody (NBP2-48300, Novus Biologicals; 1 drop per 250 μL, 20 min incubation) and visualized with Opal 690. For immune cell detection, Foxp3 was stained using an anti-Foxp3 antibody (Ab215206, Abcam; 1:100 dilution, 20 min incubation) with Opal 570, while CD161 was stained using an anti-CD161 antibody (clone C8, BioLegend; 1:100 dilution, 20 min incubation) with Opal 570. Nuclear counterstaining was performed with DAPI (1:1000 dilution, 5 min incubation). Following the final staining cycle, slides were mounted with VECTASHIELD mounting medium (Vector Laboratories, Burlingame, CA, USA). Single-color control slides were prepared using archival TAA-induced rat CCA tissues to validate antibody specificity and to compare multiplex staining patterns with conventional single-plex IHC. Multiplex immunofluorescence images were acquired using a Leica DM6000 fluorescence microscope equipped with Leica Imaging Suite software. Exposure times for each fluorochrome were manually optimized to minimize tissue autofluorescence and signal overlap. Quantitative image analysis was performed using MetaMorph software (Molecular Devices, Inc.), allowing for enumeration of PanCK-positive tumor cells, Globo H-positive cells, Foxp3-positive regulatory T cells, and CD161-positive NK cells within defined tumor regions.

### IHC Staining

The primary antibody used in this study was VK9, a monoclonal antibody against Globo H, kindly provided by Dr. Govindaswami Ragupathi (Memorial Sloan-Kettering Cancer Center, New York, NY). Antibodies against ABCG2 (sc-69988, Santa Cruz Biotechnology, USA) and Ki67 (MIB-1, Dako, USA) were used for IHC. IHC staining for Globo H, ABCG2, and Ki67 was performed on FFPE human, PDX, and rat tumor tissues using the BOND RXm stainer and Leica BOND™ Polymer Refine Detection Kit (DS9800, Leica Biosystems, Vista, CA) according to the manufacturer's instructions. Digital images were captured at 40× magnification using the Aperio Scope AT Turbo Slide Scanner (Leica Biosystems). For human and PDX samples, IHC scoring was performed by Y. Huang, who calculated the H score using the formula: 3 × percentage of strong staining + 2 × percentage of moderate staining + percentage of weak staining (range 0-300). For rat tumor tissues, IHC images were analyzed using StrataQuest software (TissueGnostics, Vienna, Austria) to quantify marker expression.

### Immunoblotting

Whole-cell lysates were prepared and immunoblotted as previously described 32. Protein samples were boiled for 5 min after mixing with protein loading buffer. Equal amounts of total protein samples were loaded onto 8-12% sodium dodecyl sulfate-polyacrylamide gel (SDS-PAGE) for electrophoresis, and the proteins were then transferred to PVDF membranes. Blots were incubated in Tris-buffered saline (TBS) blocking buffer containing 3% BSA for 1 h at room temperature, followed by overnight incubation with the respective primary antibodies, which were diluted in TBST (containing 0.1% Tween20 and 3% BSA) at 4 °C. Subsequently, blots were washed and incubated with appropriate secondary antibodies in TBST. The Western blotting results were quantified by measuring the band intensity ratios of the target genes to GAPDH using ImageJ. Antibodies for ABCG2 (GTX100437), ABCC2 (GTX23373), ABCC4 (GTX15602), PRKACA (GTX104934) and GAPDH (GTX100118) were purchased from GeneTex Inc (USA). Anti-p-CREB (#9198), CREB (#9104) were from Cell Signaling Technology (USA). TRAX (sc-271632) and A2AR (sc-32261) antibodies were from Santa Cruz Biotechnology (USA).

### Antibody-dependent Cellular Cytotoxicity (ADCC)

The ADCC assay was conducted following previously established protocols 33. Briefly, GBC cells derived from PDX were prepared at a concentration of 2 × 10^6^ cells/mL and labeled with BATDA (C136-100, PerkinElmer) as target cells. Peripheral blood mononuclear cells (PBMC) from healthy donors, harvested using Ficoll-Paque (Amersham Biosciences) density gradient, were added to the target cells (1 × 10^5^ cells/well) at the indicated effector-to-target ratios. The co-culture was incubated at 37 °C for four h in the presence or absence of VK9 (2 μg/mL). After incubation, 10 μL of the supernatant was transferred to a clear 96-well plate and mixed with 100 μL Europium solution (C135-100, PerkinElmer), followed by a 15-min incubation at room temperature. The signal was measured using a plate reader VICTOR 3 (PerkinElmer). The percentage of specific release was calculated as 100% [(experimental release - spontaneous release) / (maximal release - spontaneous release)]. All experiments were performed in triplicate.

### GBC PDX Model

The Experimental Animal Ethics Committee of Chang Gung Memorial Hospital and Chang Gung University (Taoyuan, Taiwan) approved all animal protocols used in this study (license number: CGU109-193). GBC tumor sample was obtained from a patient undergoing surgery. The tumor was cut immediately into small sections, immersed in PBS with antibiotics, and implanted subcutaneously in the flank area into 6-8 week old female NSG mice, purchased from The Jackson Laboratory (Bar Harbor, ME, USA), and maintained under specific pathogen-free conditions. After the size of the xenograft reached 1 cm in diameter, the xenograft was excised to the diameter of 0.5 cm and sub-implanted into following passages of mice. One day after subcutaneous tumor implantation was conducted in the mice, the mice were randomly divided into a control group (n = 5) and a treatment group (n = 5), receiving weekly intravenous injections of phosphate-buffered saline (PBS; pH 7.4) or Globo H antibody drug conjugate (VK9-ADC) (10 mpk), respectively, for three weeks. During the course of treatment, the tumor size was measured every week. To test the efficiency of combined treatment with Anti-Globo H antibody (VK9) and gemcitabine, the mice were divided into four groups: control (PBS), gemcitabine (Gem), VK9 (an antibody against Globo H), and a combination of gemcitabine and VK9. Tumor growth was monitored and recorded as fold change relative to the initial tumor size (day 0) over a period of 60 days. Statistical analysis was performed to assess the significance of differences in tumor growth between the groups. This study followed the ARRIVE guidelines^31^.

### Preparation of Anti-Globo H mAb-TriM-MMAE ADC

To remove galactose and sialic acid moieties from the N-glycan of anti-Globo H monoclonal antibody (mAb VK9), 50 mg of the antibody was treated with 800 units of β1,4-galactosidase (NEB, P0745L) and 1250 units of α2-3,6,8 neuraminidase (NEB, P0720L) in 1X GlycoBuffer at 37 °C for 24 h. An additional 200 units of β1,4-galactosidase was subsequently added, and the reaction was incubated at 37 °C for another 24 h to generate a G0F/G0 antibody. The glycan-modified antibody was then purified using rProtein A Sepharose Fast Flow (GE Healthcare, 17-1279-02). The purified trimannosyl-anti-Globo H mAb (45 mg) was mixed with UDP-GlcNAz (25 mg) in 1X buffer SP (25 mM MES, 10 mM MnCl2, pH 6.5) and incubated at 37 °C for 16 h in the presence of 1.5 mg rabbit MGAT-1 and 1 mg rat MGAT-2. Following incubation, the resulting anti-Globo H mAb-TriM-4Az was subjected to conjugation with DBCO-PEG3-VC-PAB-MMAE. For this process, 646 μL of 10 mM DBCO-PEG3-VC-PAB-MMAE, 634 μL of DMSO, and 2.02 mL of 25 mM MES buffer (pH 6.5) were added to the anti-Globo H-TriM-4Az solution (4.38 mL, 7.3 mg/mL). The mixture was incubated in a shaking incubator at 37°C for 18 h. After incubation, the anti-Globo H-4 (DBCO-PEG3-VC-PAB-MMAE) was concentrated, and excess linker-payload was removed using an Amicon Ultra-15 centrifugal filter with a 30 kDa NMWL. The drug-to-antibody ratio (DAR) was determined by liquid chromatography-mass spectrometry (LC-MS), and the purity of the antibody-drug conjugate (ADC) was assessed by size-exclusion chromatography high-performance liquid chromatography analysis.

### Reverse-transcription Quantitative Real-time Polymerase Chain Reaction

SNU308 and TGBC24 cells were incubated with GlcCer and GHCer at the indicated concentration and time point. We isolated total RNA using the WelPrep Cell/Tissue RNA Kit (Welgene, Daegu, Korea), following the manufacturer's instructions. We converted one microgram of total RNA, collected from GBC cells, into cDNA using the High-Capacity cDNA Reverse Transcription Kit (ref. 4368813, Thermo Fisher Scientific, NY, USA), following the manufacturer's instructions. Expression of mRNA, including ABCB1, ABCB2, ABCB4, ABCB5, ABCB6, ABCB7, ABCB11, ABCC1, ABCC2, ABCC3, ABCC4, ABCC5, ABCC6, ABCC10, ABCC11, RPM1, RPM2 and MUC4, was determined by Fast SYBR Green Master Mix (Applied Biosystems, CA, USA). Primer pairs for ABCB1 (HP200865), ABCB2 (HP208088), ABCB4 (HP229029), ABCB5 (HP218882), ABCB6 (HK200483), ABCB7 (HP207636), ABCB11 (HP207207), ABCC1 (HP208219), ABCC2 (HP200372), ABCC3 (HP207241), ABCC4 (HP233730), ABCC5 (HP233064), ABCC6 (HP226921), ABCC10 (HP216443), ABCC11 (HP216047), RRM1 (HK206462), RRM2 (HP203086), MUC4 (HP232719), ENT1 (HP225775), DCK (HP200729), SLC28A3 (HP214376), BRCA1 (HP210038), BRCA2 (HP200045), RAD51 (HP206489), BCL2 (HP200598), BCL2L1 (HP234144), TWIST1 (HP200446), SNAI1 (HP209016), SNAI2 (HP206658) and GAPDH (HP205798) were purchased from OriGene (Rockville, MD, USA). All PCR reactions were conducted on an Applied Biosystems 7500 Fast Real-Time PCR System with the following reaction conditions: pre-incubation at 95 °C for 10 s to activate HotStart Taq DNA polymerase, followed by 40 cycles of denaturation at 95 °C for 15 s and annealing/extension at 60 °C for 1 min. In each run, nuclease-free water was used as a negative control to replace cDNA in PCR amplification. Glyceraldehyde-3-phosphate dehydrogenase (GAPDH) served as the endogenous control, and 10 nanograms of cDNA sample were used for the quantitative real-time PCR (qPCR) reaction, following the manufacturer's instructions. The fluorescent signals were analyzed using 7500 Software v2.06, and relative mRNA expression was calculated using the △△Ct method 34.

### Matrix-assisted Laser Desorption Ionization-Time of Flight (MALDI-TOF) Analysis of Permethylated GSLs

GSLs were isolated from GBC PDX tissues following established protocols 35. In brief, approximately 25 mg of frozen tissue was ground and extracted using a methanol-chloroform mixture. The resulting extract was centrifuged, and the supernatant was collected. This process was repeated three times to maximize yield. The combined supernatants were subjected to anion-exchange chromatography to separate neutral and acidic GSL fractions. Permethylated GSLs were then mixed with 2,5-dihydroxybenzoic acid (DHB, Sigma-Aldrich, St. Louis, MO, USA) as the matrix. Mass spectra were recorded using a SCIEX 5800 TOF/TOF system in positive-ion mode, with spectra acquired from 4000 accumulated laser shots using a random sampling method 36.

### Measurement of Human anti-Globo H Antibody Titer using ELISA Assay

Serum samples from the patient were collected at multiple time points (weeks 0, 2, 6, 8, 12, and 16) to assess the levels of anti-Globo H IgG and IgM antibodies using an ELISA assay. ELISA plates (Thermo Scientific, 442404) were coated overnight with 40 ng of GHCer. After blocking, patient serum samples were diluted and applied to the plates. Anti-Globo H antibodies (IgG and IgM) were detected using HRP conjugated secondary antibodies specific for human IgG (anti-human IgG HRP) (Jackson ImmunoResearch Laboratories, Inc, 109-035-003) and IgM (anti-human IgM HRP) (Jackson ImmunoResearch Laboratories, Inc, 109-035-129). The optical density (OD) values from Globo H-coated wells were subtracted from the OD values of uncoated control wells to account for background noise. The cutoff value was calculated by adding 0.1 to the mean OD value of the secondary antibody-only control. Antibody titers were defined as the highest serum dilution that produced an absorbance reading equal to or above the cutoff value.

### Rhodamine 123 Efflux Assay

Drug efflux activity was assessed using a Rhodamine 123 (Rh123) efflux assay. SNU308 and TGBC24 GBC cells were treated with GlcCer or GHCer for 72 h, with the anti-Globo H monoclonal antibody VK9 added during GHCer treatment where indicated. After treatment, cells were incubated with Rh 123 (1 μM) at 37 °C for 30 min to allow intracellular dye loading. Cells were then washed with PBS to remove excess dye and resuspended in dye-free complete medium to allow efflux. Following the efflux period, cells were collected and analyzed by flow cytometry. Unstained cells were used to define background fluorescence. Intracellular Rh123 fluorescence intensity was measured, and changes in fluorescence distribution were interpreted as alterations in drug efflux activity.

### Statistical Analysis

The data were presented as mean ± SD, counts, and percentages. Group differences were assessed using the Mann-Whitney U test and Pearson Chi-square test. Survival curves were analyzed using the Kaplan-Meier method with the log-rank test, conducted through Prism 6.0 (GraphPad Software, La Jolla, CA, USA). To identify independent prognostic factors in GBC, Cox proportional-hazards regression analysis was performed using SPSS software (SPSS Inc., Chicago, IL, USA).

## Results

### ABCG2 Upregulation by GHCer Contributes to GR in GBC Cells

**Figure 1:**
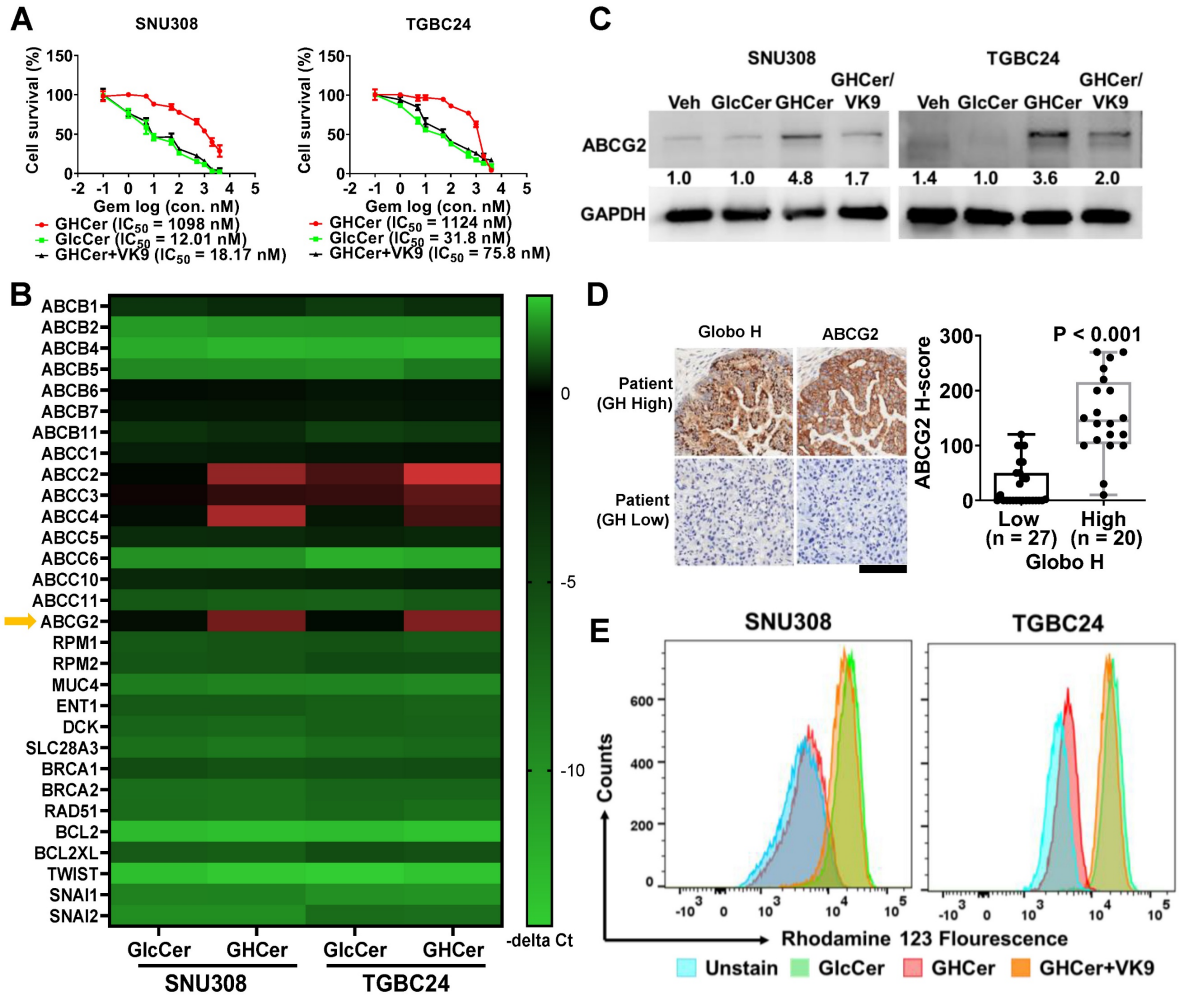
** Upregulation of ABCG2 by GHCer in GBC Cells.** (A) Dose-response curves showing the effects of increasing concentrations of gemcitabine on the survival of SNU308 and TGBC24 GBC cells in the presence of GHCer (30 μM), GlcCer, or GHCer combined with the anti-Globo H monoclonal antibody VK9 (10 μg/mL). Cell viability was assessed at 72 h, and the half-maximal inhibitory concentration (IC₅₀) values for each condition are indicated. (B) Heatmap illustrating relative mRNA expression changes of ABC transporters and other GR-related genes, including drug efflux transporters, nucleoside transporters, DNA damage response-associated genes, and survival-related genes, in SNU308 and TGBC24 cells treated with GHCer (30 μM) for 72 h compared with GlcCer-treated controls. Gene expression levels were determined by quantitative RT-PCR and are presented as -ΔCt values. ABCG2 is highlighted by a yellow arrow. (C) Western blot analysis of ABCG2 protein expression in SNU308 and TGBC24 cells treated with GHCer (30 μM), GHCer in combination with VK9 (10 μg/mL), or GlcCer (control) for 72 h. GAPDH was used as a loading control. Numbers shown below the bands indicate densitometric ratios normalized to GAPDH and expressed relative to the GlcCer control condition. (D) Representative IHC images showing Globo H and ABCG2 expression in tumor tissues from patients with advanced GBC stratified into Globo H-high (H-score ≥ 80) and Globo H-low (H-score < 80) groups. The box-and-whisker plot summarizes the association between Globo H and ABCG2 expression levels based on H-scores, demonstrating a significant positive correlation (P < 0.001). Scale bars, 60 μm. (E) Representative flow cytometry histograms showing intracellular Rh123 fluorescence in SNU308 and TGBC24 cells following treatment with GlcCer or GHCer, in the presence or absence of the anti-Globo H antibody VK9. Cells were treated with GlcCer or GHCer for 72 h, with VK9 added during GHCer treatment where indicated, and subsequently incubated with Rh123 (1 μM) for 30 min. After washing, cells were subjected to efflux analysis by flow cytometry. A leftward shift of the fluorescence peak indicates enhanced drug efflux activity, whereas a rightward shift reflects reduced efflux. Unstained cells are shown as negative controls.

To investigate the mechanisms underlying the poor prognostic impact of Globo H expression in GBC, we examined the possible contribution of GHCer to gemcitabine sensitivity in GBC cell lines, SNU308 and TGBC24. Incubation of these two cell lines with GHCer significantly increased the IC_50_ values of gemcitabine (12.01 vs. 1098 nM for SNU308; 31.8 vs 1124 nM for TGBC24), which were reduced to 18.17 and 75.8 nM, respectively, by neutralizing GHCer with mAb VK9 (Figure 1A). Given that MUC4, RRM1, RRM2, and ABC transporters are associated with GR 37-39, we next conducted a heatmap analysis of quantitative polymerase chain reaction (qPCR) of these genes in two GBC cell lines, SNU308 and TGBC24, in response to incubation with GHCer. As shown in Figure 1B, significant upregulation of ABCC2, ABCC4, and ABCG2 mRNA expression was observed, and western blot analysis showed 4.8 and 3.6 fold increase in ABCG2 protein levels, respectively, after GHCer treatment, which was attenuated by the addition of mAb VK9 (Figure 1C). In contrast, the expression levels of ABCC2 and ABCC4 proteins were not discernible in SNU308 or TGBC24 cells, with no significant changes following GHCer treatment (Figure S1). Immunohistochemical staining of 47 GBC tissues further demonstrated a significant correlation between high Globo H expression (H score ≥ 80) and elevated ABCG2 levels (P < 0.001) (Figure 1D). To determine whether GHCer-induced ABCG2 upregulation is functionally associated with enhanced drug efflux activity, we performed an Rh123 efflux assay in GBC cells. SNU308 and TGBC24 cells were treated with GlcCer or GHCer for 72 h, followed by incubation with Rh123 (1 μM) for 30 min and subsequent efflux analysis by flow cytometry. Compared with GlcCer-treated controls, GHCer-treated cells exhibited a pronounced leftward shift in Rh123 fluorescence intensity, indicating increased efflux activity. Importantly, co-treatment with the anti-Globo H monoclonal antibody VK9 partially reversed the GHCer-induced decrease in intracellular Rh123 accumulation, suggesting that the enhanced efflux activity is dependent on Globo H-associated signaling (Figure 1E). These findings suggest that ABCG2 upregulation by GHCer contributes to drug resistance in GBC, positioning it as a key target for overcoming chemoresistance.

### Upregulation of ABCG2 Expression via A2AR/cAMP/PKA Signaling Pathway in GBC Cells

**Figure 2:**
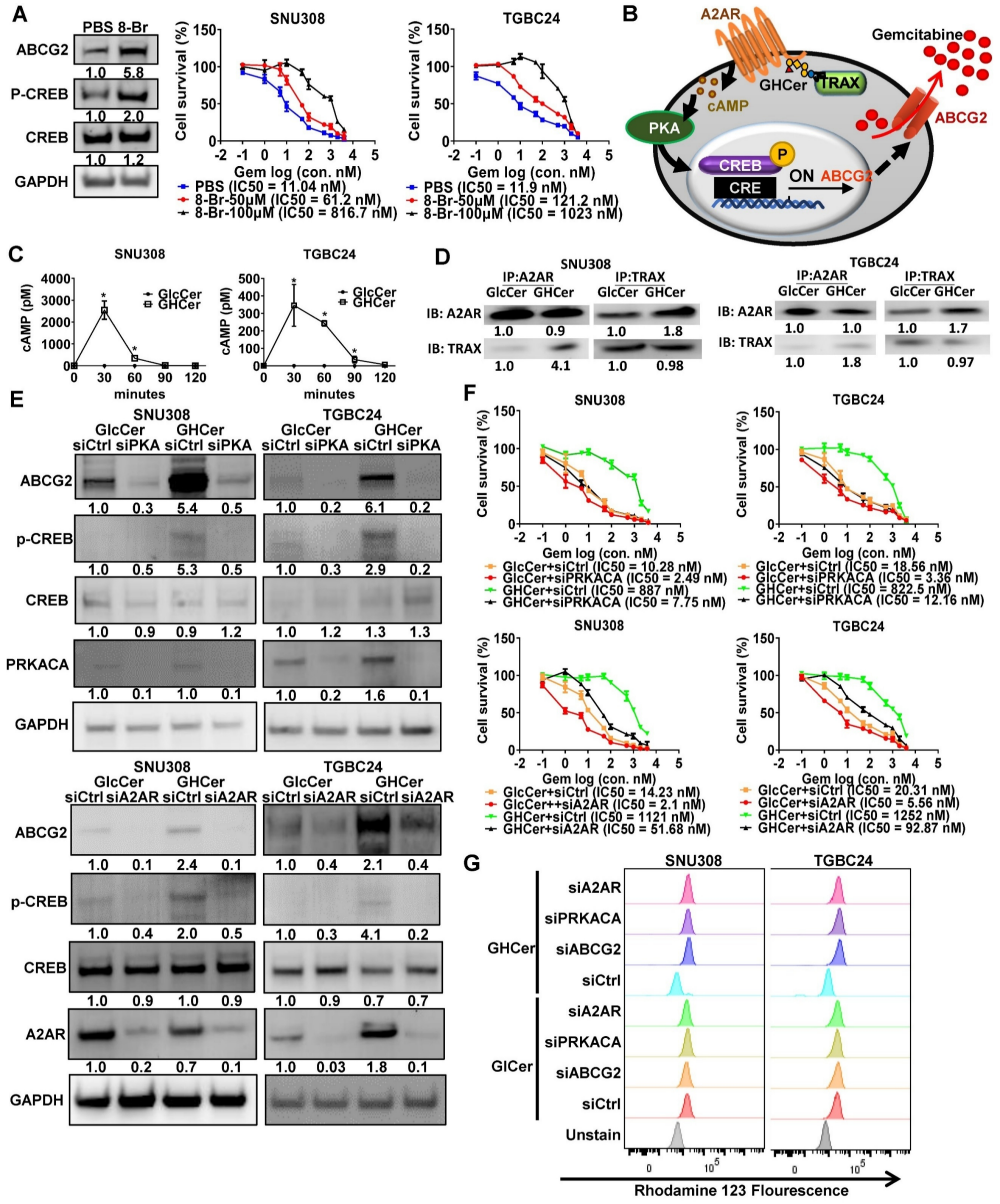
** Upregulation of ABCG2 via A2AR/cAMP/PKA Signaling in GBC Cells.** (A) Left panel: Western blot analysis of ABCG2, phosphorylated CREB (p-CREB), and total CREB in SNU308 cells treated with the membrane-permeable cAMP analog 8-Br-cAMP (100 μM) for 48 h, demonstrating activation of the cAMP/PKA signaling axis. GAPDH was used as a loading control. Right panel: Cell viability assays of SNU308 and TGBC24 cells treated with increasing concentrations of gemcitabine for 72 h in the presence or absence of 8-Br-cAMP (50 or 100 μM). Dose-response curves were generated and the half-maximal inhibitory concentration (IC₅₀) values of gemcitabine were calculated. (B) Schematic illustration of the proposed molecular mechanism whereby GHCer interacts with the A2AR-TRAX complex, leading to activation of the cAMP/PKA signaling cascade, phosphorylation of CREB, transcriptional upregulation of ABCG2, and enhanced drug efflux-mediated chemoresistance. (C) Intracellular cAMP levels (pM per 10⁶ cells) in SNU308 and TGBC24 cells treated with GHCer (30 μM) or GlcCer (30 μM) for the indicated time points (30, 60, 90, and 120 min). cAMP concentrations were quantified using a competitive ELISA assay. GHCer treatment significantly increased intracellular cAMP levels in both cell lines, with peak induction observed at 30 min (*p < 0.05 vs. GlcCer). (D) Co-immunoprecipitation analysis demonstrating the association between A2AR and TRAX in GBC cells. SNU308 and TGBC24 cells were treated with GHCer (30 μM) or GlcCer (30 μM) for 24 h, followed by immunoprecipitation with anti-A2AR or anti-TRAX antibodies. Immunoprecipitates were subjected to western blotting and probed with anti-TRAX or anti-A2AR antibodies, respectively. Enhanced A2AR-TRAX complex formation was observed in GHCer-treated cells compared with GlcCer controls. (E) Western blot analysis of ABCG2, p-CREB, total CREB, PRKACA (PKA catalytic subunit), and A2AR in SNU308 and TGBC24 cells transfected with siPRKACA (siPKA) or siA2AR for 24 h, followed by treatment with GHCer (30 μM) for 72 h. GAPDH served as a loading control. (F) Cell viability assays were performed in parallel. Cells were transfected with siRNA for 24 h, then treated with GHCer (30 μM) or GlcCer (30 μM) in combination with increasing concentrations of gemcitabine for 72 h. IC₅₀ values were calculated to assess the impact of A2AR or PRKACA knockdown on GHCer-induced GR. (G) Representative flow cytometry histograms showing intracellular Rh123 fluorescence in SNU308 and TGBC24 GBC cells. Cells were transfected with control siRNA (siCtrl), siRNA targeting A2AR (siA2AR), PRKACA (siPRKACA), or ABCG2 (siABCG2) for 72 h, followed by treatment with GHCer or GlcCer for an additional 48 h. Cells were then incubated with Rh123 (1 μM) for 30 min at 37 °C, washed, and analyzed by flow cytometry. Unstained cells were included as negative controls. A leftward shift of the Rh123 fluorescence peak indicates reduced intracellular dye accumulation consistent with enhanced drug efflux activity, whereas a rightward shift reflects increased intracellular Rh123 retention.

ABCG2 expression is regulated by the cAMP/PKA signaling pathway 17. Indeed, treatment with 8-Br-cAMP (a cAMP analog) increased ABCG2 and p-CREB expression in SNU308 cells and enhanced GR in both SNU308 and TGBC24 cells in a concentration-dependent manner, as evidenced by increased IC_50_ values (Figure 2A). Thus, the cAMP/PKA pathway plays an important role in GR in SNU308 and TGBC24 cells. Based on our previous studies showing that GHCer enhances cAMP/PKA signaling through A2AR activation 20, we hypothesized that GHCer modulates ABCG2 expression via A2AR/cAMP/PKA axis (Figure 2B). In both SNU308 and TGBC24 cell lines, incubation with GHCer significantly elevated intracellular cAMP levels, peaking 30 min post-treatment (Figure 2C). Co-immunoprecipitation and immunoblot analyses revealed that GHCer enhanced the interaction between A2AR and TRAX in these cells (Figure 2D). Western blot analysis further confirmed the increased expression of ABCG2 and phosphorylated CREB (p-CREB) following GHCer treatment compared to controls. When GHCer was combined with either H89 (PKA inhibitor) or ZM241385 (A2AR antagonist), the upregulation of ABCG2 and p-CREB was significantly attenuated in SNU308 and TGCB24 cells (Figure S2A), suggesting that the effect of GHCer on ABCG2 expression was dependent on A2AR/cAMP/PKA signaling pathway. Furthermore, GHCer-induced GR was significantly reduced by co-treatment with H89 or ZM241385 (Figure S2B).

To further confirm the role of A2AR and PRKACA in the GHCer-induced signaling pathway leading to ABCG2 expression, siRNA-mediated knockdown experiments were conducted in GBC cell lines. The efficiency of knockdown in SNU308 and TGBC24 cell lines ranged from 0.2 to 0.4 fold for A2AR (Figure S3A-B) and 0.3 to 0.5 fold for PRKACA (Figure S3D-E). Knockdown of PKA or A2AR significantly decreased GHCer-induced ABCG2 expression and reversed GR, as confirmed by the significantly reduced IC_50_ values (Figure 2E-F) in both cell lines. To further determine whether the chemoresistant effect of GHCer was specific to gemcitabine or extended to other drugs, we examined the response of TGBC24 and SNU308 cells to cisplatin. GHCer pretreatment increased cisplatin resistance in both cell lines, whereas the anti-Globo H antibody VK9 partially restored cisplatin sensitivity (Figure S4). These results suggest that GHCer confers a broader chemoresistant phenotype, potentially mediated through ABCG2 activation and A2AR/PKA signaling. To further determine whether the GHCer-associated increase in drug efflux activity is mediated through the A2AR-PKA-ABCG2 signaling axis, we performed siRNA-mediated knockdown experiments followed by an Rh123 efflux assay (Figure 2G). SNU308 and TGBC24 cells were transfected with siA2AR, siPRKACA, or siABCG2, with a non-targeting siRNA used as control (siCtrl). Following gene silencing, cells were treated with GHCer or GlcCer and subjected to Rh123 efflux analysis by flow cytometry. In siCtrl-transfected cells, GHCer treatment induced a pronounced leftward shift of the Rh123 fluorescence peak, consistent with enhanced efflux activity. In contrast, knockdown of A2AR, PRKACA, or ABCG2 markedly attenuated the GHCer-induced Rh123 efflux, resulting in a rightward shift indicative of increased intracellular dye retention (Figure 2G). Quantitative RT-PCR confirmed efficient suppression of A2AR, PRKACA, and ABCG2 expression following siRNA transfection (Figure S5). Notably, silencing of ABCG2 produced the most prominent reversal of the GHCer-associated efflux phenotype, supporting a direct role for ABCG2 as the downstream effector. These findings demonstrate that GHCer-enhanced drug efflux activity is functionally dependent on the A2AR-cAMP/PKA-ABCG2 signaling pathway, providing mechanistic evidence that this axis contributes to GHCer-associated chemoresistance in GBC cells. Collectively, these results suggest that GHCer promotes ABCG2 expression and GR in GBC cells through A2AR/cAMP/PKA signaling pathway.

### Targeting Globo H to dampen A2AR/cAMP/PKA Signaling and Overcome Chemoresistance in GBC

**Figure 3:**
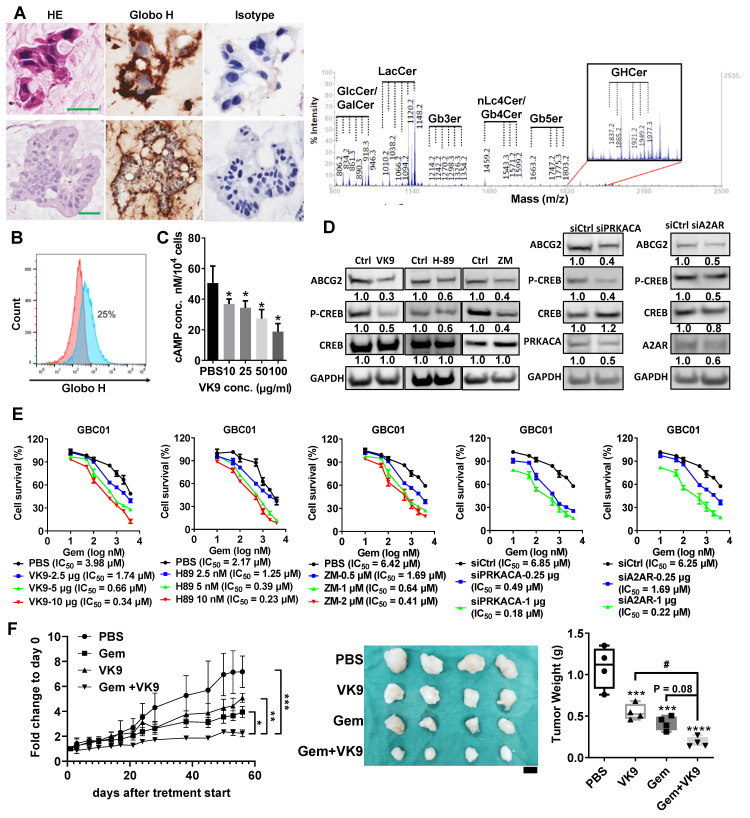
** Inhibition of GHCer/A2AR/PKA signaling reduces ABCG2 expression and enhances chemosensitivity in GBC01 cells.** (A) Upper panels: Immunohistochemical staining of Globo H in FFPE human GBC tumor tissues using the anti-Globo H monoclonal antibody VK9. Lower panels: Immunohistochemical detection of Globo H in GBC PDX tumors. Scale bars, 60 μm. Right panels: Mass spectrometric analysis of glycolipid extracts isolated from GBC PDX tumors confirming the presence of GHCer and related glycosphingolipid species. (B) Cell-surface expression of Globo H in GBC01 cells analyzed by flow cytometry following staining with mAb VK9. Murine IgG3 was used as an isotype-matched negative control. (C) Intracellular cAMP levels in GBC01 cells following treatment with mAb VK9 at the indicated concentrations (10-100 μg/mL) for 24 h. cAMP levels were quantified using a competitive ELISA assay. Data are presented as mean ± SEM (*P < 0.05 vs. control). (D) Left panels: Western blot analysis of ABCG2, p-CREB, total CREB, and GAPDH in GBC01 cells treated with mAb VK9 (10 μg/mL), the PKA inhibitor H89 (10 nM), the A2AR antagonist ZM241385 (ZM) (2 μM), or Ctrl (DMSO) for 48 h. Right panels: Western blot analysis of ABCG2, p-CREB, total CREB, PRKACA, A2AR, and GAPDH in GBC01 cells 72 h after transfection with siPRKACA, siA2AR, or non-targeting siRNA (siCtrl). (E) Cell viability assays of GBC01 cells treated with increasing concentrations of gemcitabine for 72 h in combination with mAb VK9, H89, or ZM241385 (ZM) at the indicated concentrations. For siRNA experiments, cells were transfected with siPRKACA or siA2AR for 48 h prior to gemcitabine treatment. Dose-response curves were generated and half-maximal inhibitory concentration (IC₅₀) values of gemcitabine were calculated for each condition. (F) *In vivo* therapeutic evaluation in a GBC PDX model. NSG mice bearing established GBC PDX tumors were randomized to receive PBS, gemcitabine (Gem, 10 mg/kg), mAb VK9 (10 mg/kg), or the combination of Gem and VK9 by weekly administration for four weeks (n = 4 per group). Left: Tumor growth curves during treatment; statistical significance was assessed by one-way ANOVA (*P < 0.05, **P < 0.01, ***P < 0.001 vs. PBS). Middle: Representative images of excised tumors at week 8 (scale bar, 0.5 cm). Right: Tumor weights measured at week 8; statistical significance was determined by one-way ANOVA (***P < 0.001, ****P < 0.0001 vs. PBS). In addition, direct comparison between Gem and Gem + VK9 groups was performed using a two-tailed Student's t-test, yielding a p value of 0.03.

To address the critical challenge of chemoresistance in GBC, we investigated the therapeutic potential of targeting Globo H, using a PDX model. This model was established from the surgical specimen of a 78-year-old male patient with recurrent GBC. A mucinous adenocarcinoma developed within two months after tumor implantation in NSG mice. Both PDX and patient tumors showed strong Globo H expression by IHC and the presence of GHCer and related glycosphingolipids by mass spectrometry (Figure 3A). A cell line, GBC01 was subsequently generated from this PDX tumor. Flow cytometry revealed the surface expression of Globo H in approximately 25% of GBC01 cells (Figure 3B). To investigate whether blocking GHCer-induced A2AR/cAMP/PKA signaling could reduce ABCG2 expression, we treated GBC01 cells with mAb VK9 at various concentrations for 24hrs. Intracellular cAMP levels were significantly reduced by mAb VK9 in a dose-dependent manner (*P < 0.05; Figure 3C). Western blot analysis showed that mAb VK9 reduced ABCG2 and p-CREB expression without affecting CREB levels, similar to H89 and ZM241385. These findings indicate that blocking Globo H or interfering with the A2AR/cAMP/PKA pathway can downregulate ABCG2 (Figure 3D). Additionally, knockdown of PRKACA (the catalytic subunit of PKA) or A2AR using siRNA (Figure S3C, S3F) led to comparable reductions in ABCG2 and p-CREB expression in GBC01 cells (Figure 3D). Cell viability assays revealed that GBC01 cells exhibited increased sensitivity to gemcitabine when treated with mAb VK9, H89, ZM241385, or siRNAs targeting PRKACA or A2AR in a dose-dependent manner, as evidenced by the reduced IC_50_ values compared to controls (Figure 3E). Next, in the GBC PDX model, combined treatment with mAb VK9 and gemcitabine markedly inhibited tumor growth as assessed by tumor volume, compared with control or either treatment alone. Final tumor weight at sacrifice also trended lower in the Gem + VK9 group compared with Gem alone. The one-way ANOVA across all treatment groups yielded p = 0.08, whereas a pairwise t-test specifically comparing Gem and Gem + VK9 showed a significant difference (p = 0.03), consistent with the observed reduction in tumor volume (Figure 3F). Throughout the treatment course, no significant changes in body weight were observed across all groups (Figure S6). These findings highlight that Globo H inhibition or disruption of A2AR/cAMP/PKA pathway effectively reduced ABCG2 expression and enhanced the chemosensitivity of GBC cells.

### Clinical Efficacy of OBI833/OBI821 in a GBC Patient with Chemotherapy Resistance

**Figure 4:**
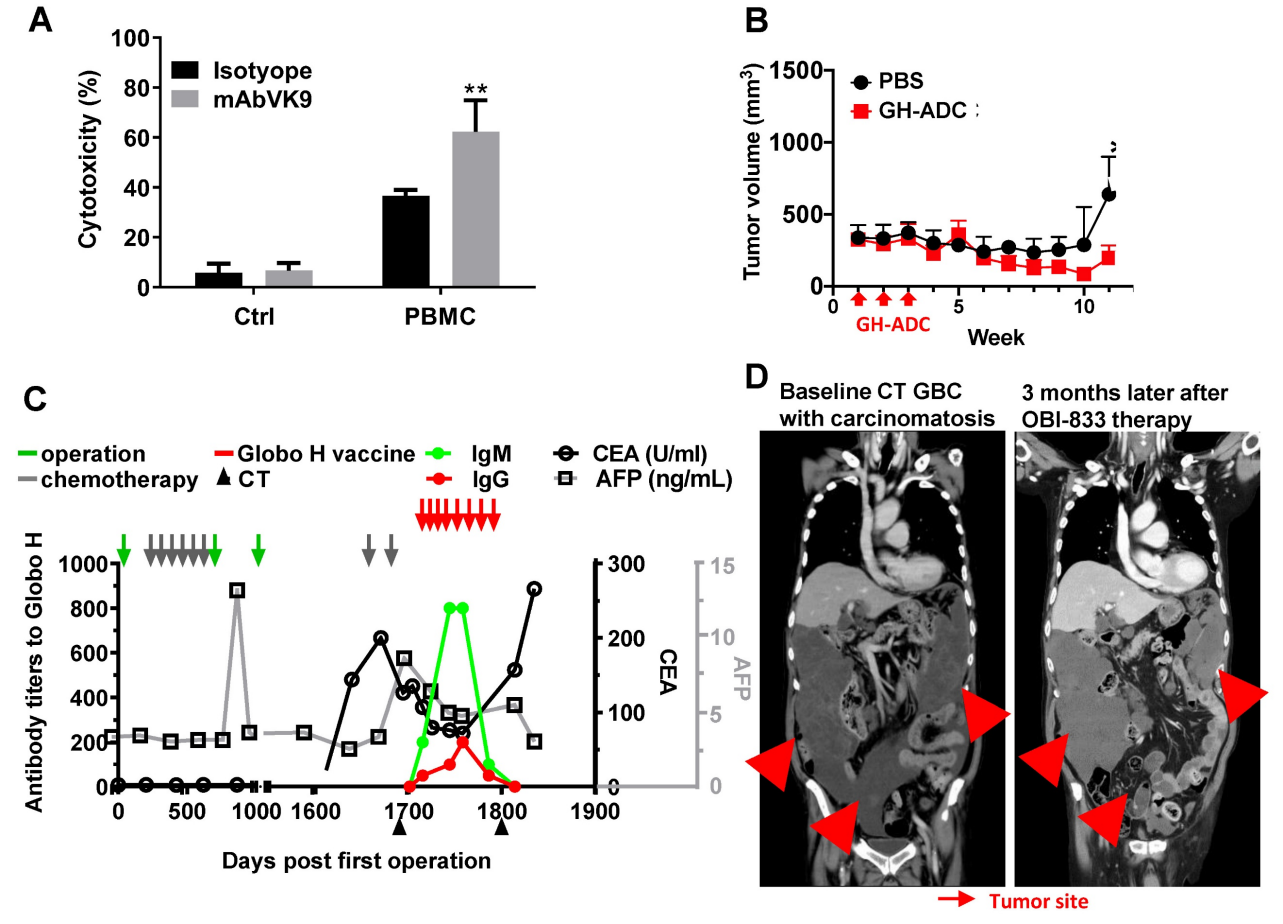
***In vitro* and *in vivo* anti-cancer efficacy of Globo H targeted therapies in a patient with recurrent GBC and his tumor cells.** (A) ADCC mediated by anti-Globo H monoclonal antibody VK9 in GBC01 cells. GBC01 target cells were incubated with mAb VK9 (2 μg/mL) or murine IgG3 isotype control, and co-cultured with human peripheral blood mononuclear cells (PBMCs) as effector cells at an effector-to-target (E:T) ratio of 10:1. ADCC was quantified using a BATDA-based fluorescence cytotoxicity assay by measuring fluorescence-based specific release. “Ctrl” indicates target cells incubated without PBMCs, whereas “PBMC” indicates co-culture conditions. Data are presented as mean ± SEM. **P < 0.01 versus isotype control. (B) *In vivo* antitumor efficacy of a Globo H antibody-drug conjugate (Globo H-ADC; anti-Globo H antibody conjugated to MMAE) in a GBC PDX model. Tumor-bearing mice were randomized to receive PBS (vehicle control) or Globo H-ADC (10 mg/kg; n = 5 per group). Globo H-ADC was administered intravenously after tumor implantation, with three weekly doses (indicated by red arrows). Tumor volumes were measured longitudinally and plotted over time. Globo H-ADC significantly suppressed tumor growth compared with PBS (*P < 0.05; statistical test as described in Methods). (C) Longitudinal clinical monitoring of serum tumor markers and vaccine-induced humoral immune responses in a GBC patient receiving compassionate use of Globo H vaccine therapy (OBI833/OBI821). Serial measurements of anti-Globo H antibody titers (IgM and IgG) and serum tumor markers (CEA [U/mL] and AFP [ng/mL]) are shown over time (days post first operation). Major clinical interventions—including surgery, chemotherapy cycles, CT imaging assessments, and administration of the Globo H vaccine—are annotated with arrows as indicated in the legend. Antibody titers and tumor markers were tracked to evaluate immune response kinetics and clinical disease dynamics during treatment. (D) Representative contrast-enhanced CT images of the same patient at baseline and 3 months after initiation of OBI833/OBI821 therapy. Baseline imaging demonstrated multifocal recurrent disease with carcinomatosis (red arrows). Follow-up imaging showed resolution of a pelvic lesion with slight enlargement of a right upper quadrant lesion (red arrows), consistent with SD at the time of assessment. Tumor sites are indicated by arrows.

Our findings suggest that targeting Globo H may hold therapeutic potential for patients with recurrent GBC. Importantly, immunohistochemical analysis of the tumor tissues obtained from the patient before and after chemotherapy demonstrated a significant elevation in Globo H expression (H-score: 80 pre-chemotherapy, 160 post-chemotherapy) and a moderate increase in ABCG2 expression (H-score: 100 pre-chemotherapy, 180 post-chemotherapy) (Figure S7). To support the compassionate IND for treating this patient with Globo H vaccine (OBI833/OBI821), we assessed the *in vitro* ADCC activity of mAb VK9 on the tumor cells GBC01. In the presence of peripheral blood mononuclear cells, mAb VK9 antibody induced significantly greater cytotoxicity in GBC01 cells than the isotype control (62.3% vs. 36.7% lysis, P = 0.003) (Figure 4A). *In vivo*, administration of Globo H -ADC, an anti- Globo H antibody conjugated with MMAE, significantly inhibited tumor growth in the PDX model derived from this patient (Figure 4B). Based on these data, the patient received OBI833/OBI821 weekly × 4, followed by every two weeks × 4. Post-treatment serological analysis revealed a gradual and significant rise in his antibody titers against Globo H (IgM and IgG), along with a noticeable decline in his serum levels of carcinoembryonic antigen (CEA) from 200 to 72 ng/mL and alpha-fetoprotein (AFP) from 8.5 to 4.7 ng/mL (Figure 4C). Post-treatment computed tomography (CT) imaging at 3 months demonstrated a heterogeneous radiologic response. Among the three large measurable tumor masses (indicated by arrows in Figure 4D), the inferior pelvic lesion completely resolved, the left upper quadrant reduced in size, whereas the largest right upper quadrant mass increased slightly. Overall, these findings are consistent with a mixed radiologic response with stable disease.

### Combination Therapy with OBI833/OBI821 and Gemcitabine Enhances Anti-Tumor Efficacy in a Rat CCA Model

**Figure 5:**
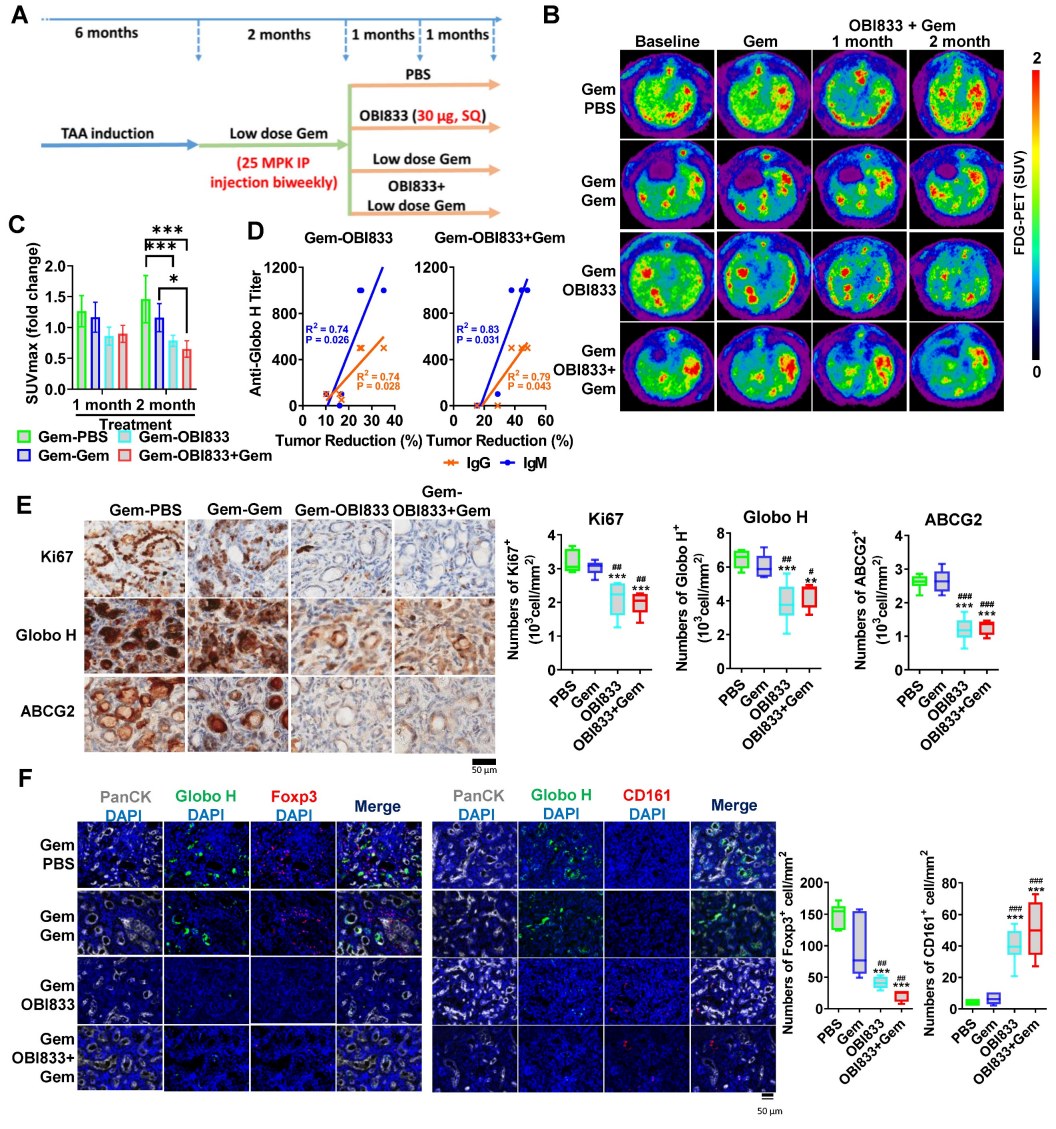
** Evaluation of combination of OBI833 and gemcitabine in TAA-induced rat CCA model.** (A) Experimental timeline and treatment regimen for the TAA-induced CCA rat model. Male Sprague-Dawley rats were administered TAA for 6 months to induce CCA. After tumor establishment, rats received low-dose gemcitabine (Gem, 25 mg/kg, intraperitoneal injection, once every 2 weeks) for 2 months. Rats were then randomized into four treatment groups (n = 6 per group): (i) Gem-PBS (continued low-dose Gem + PBS), (ii) Gem-Gem (continued low-dose Gem alone), (iii) Gem-OBI833 (continued low-dose Gem + OBI833/OBI821), and (iv) Gem-OBI833+Gem (continued low-dose Gem + OBI833/OBI821 + additional Gem as indicated in the schema). The Globo H vaccine formulation OBI833/OBI821 (OBI833 30 μg + OBI821 100 μg) was administered subcutaneously (SQ) once weekly for 2 months, consistent with the treatment window shown. FDG-PET scans were performed at baseline (prior to the second-stage treatments) and at 1 month and 2 months after initiation of the indicated treatment regimens to monitor tumor metabolic activity. (B) Representative axial FDG-PET images from each treatment group at baseline, after Gem, and at 1 and 2 months following treatment initiation. Images are displayed using a consistent pseudocolor scale representing standardized uptake value (SUV). (C) Quantification of PET-derived SUVmax fold change at 1 month and 2 months relative to baseline for each treatment group. Data are presented as mean ± SEM. Statistical comparisons were performed using one-way ANOVA (with post hoc multiple-comparison testing as described in Methods). ***P < 0.001 for Gem-OBI833 and Gem-OBI833+Gem compared with Gem-PBS at the 2-month time point; *P < 0.05 for Gem-OBI833+Gem compared with Gem-Gem at the 2-month time point. (D) Serum anti-Globo H IgM and IgG titers measured by ELISA during treatment. Antibody titers were plotted against the percentage of tumor reduction, and linear regression analysis was performed to evaluate correlations between humoral immune responses and treatment-associated tumor reduction (P < 0.05 )(E) IHC staining of CCA tumor tissues for Ki67, Globo H, and ABCG2 following treatment. Representative images are shown for each group (scale bar, 50 μm). Quantification of Ki67⁺ cells, Globo H⁺ cells, and ABCG2⁺ cells is presented as box plots. Statistical significance was assessed by one-way ANOVA: **P values versus Gem-PBS are indicated as **P < 0.01 and ***P < 0.001; comparisons versus Gem-Gem are indicated as ^#^P < 0.05, ^##^P < 0.01, and ^###^P < 0.001. (F) Opal multiplex immunofluorescence analysis of tumor sections. Tumor epithelium was identified by PanCK, and Globo H expression was co-visualized with immune markers Foxp3 (Treg marker) and CD161 (NK cell marker), with nuclei counterstained by DAPI. Representative images for each treatment group are shown (scale bar, 50 μm), including merged panels. Quantification of Foxp3⁺ and CD161⁺ cells (cells/mm²) is shown as box plots. Statistical comparisons were performed using one-way ANOVA: ***P < 0.001 versus Gem-PBS; ^##^P < 0.01 and ^###^P < 0.001 versus Gem-Gem.

To further evaluate the potential of targeting Globo H to enhance gemcitabine sensitivity* in vivo*, we utilized a TAA-induced rat CCA model that expresses Globo H 29. After CCA induction, rats were treated with low-dose gemcitabine for 2 months and then randomized to receive either PBS, gemcitabine, OBI833/OBI821 40 or a combination of the latter two (Figure 5A). PET scan analysis revealed distinct changes in the different treatment groups (Figure 5B). Rats treated with the combination of gemcitabine and OBI833/OBI821 exhibited a significant reduction in maximum standardized uptake value (SUVmax) compared to those treated with gemcitabine alone (P = 0.03) and PBS group (P = 0.002). Although the gemcitabine group did not differ from the PBS group, treatment with OBI833/OBI821 alone showed greater tumor reduction than the PBS group (P = 0.007), but not gemcitabine alone (P = 0.13) (Figure 5C). To ascertain the contribution of anti-Globo H immune responses to tumor reduction, we measured serum IgG and IgM titers in each rat treated with OBI833/OBI821 using ELISA and correlated with tumor reduction assessed by PET imaging. Linear regression analysis demonstrated a significant positive correlation between anti-Globo H IgG/IgM titers and tumor reduction in both Gem-OBI833 (IgG: R² = 0.74, P = 0.028; IgM: R² = 0.74, P = 0.026) and Gem-OBI833+Gem (IgG: R² = 0.79, P = 0.043; IgM: R² = 0.83, P = 0.031) groups, suggesting that induction of anti-Globo H IgG/IgM is crucial for tumor regression (Figure 5D). Consistent with these results, IHC analysis showed significantly lower Ki67 staining in the combination group, indicating reduced proliferation, along with fewer Globo H- and ABCG2-expressing cells compared to gemcitabine alone or control (Figure 5E). These findings suggest that the combination of OBI833/OBI821 and gemcitabine not only enhances anti-tumor efficacy but also mitigates drug resistance in GBC.

We further analyzed immune cell profiles in the CCA tumor microenvironment in rats. Immunofluorescence staining showed a significant reduction of Foxp3-positive Tregs in both the OBI833 monotherapy (Gem-OBI833; P < 0.001) and combination therapy (Gem-OBI833+Gem; P < 0.001) groups compared to the Gem-PBS group. Similar reductions were also observed when compared to the Gem-Gem group (P < 0.01 for both Gem-OBI833 and Gem-OBI833+Gem) (Figure 5F). In contrast, immunofluorescence staining showed that CD161-positive NK cells were significantly increased in both the OBI833 monotherapy group (Gem-OBI833, P < 0.001) and the combination therapy group (Gem-OBI833+Gem, P < 0.001) compared to the Gem-PBS group (Figure 5F). Similarly, a significant increase in CD161-positive cells was observed in these groups compared to the Gem-Gem group (P < 0.001). This shift in the immune cell population, with decreased Tregs and increased NK cells, highlights the immunomodulatory effects of OBI833/OBI821 and the combination therapy, further contributing to the enhanced anti-tumor efficacy. Such reshaping of the tumor microenvironment suggests that targeting Globo H not only inhibits tumor proliferation but also reprograms the immune landscape to favor anti-tumor responses.

Based on the preclinical data described above, we initiated a clinical trial combining a Globo H vaccine (OBI833/821) with chemotherapy in patients with advanced bile duct cancer who have at least achieved disease stabilization after four courses of first-line chemotherapy. Preliminary findings are encouraging.

### Prognostic Value of Globo H Expression in Advanced GBC and Its Clinical Implications

**Figure 6:**
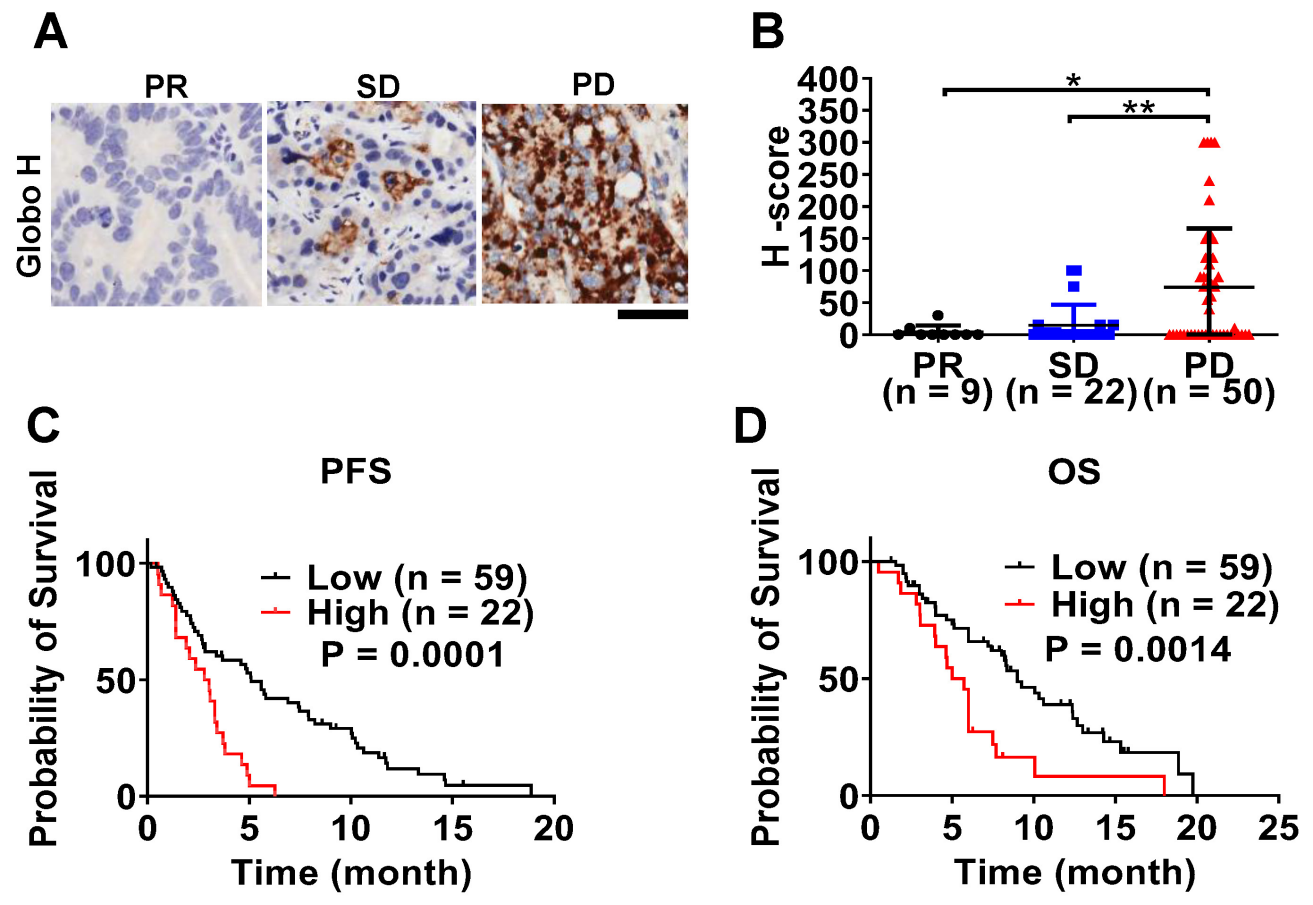
** Correlation of Globo H expression with clinical outcomes in patients with advanced GBC.** (A) Representative IHC staining of Globo H in FFPE tumor specimens from GBC patients stratified by treatment response to gemcitabine-based chemotherapy, including PR, SD, and PD. Representative images illustrate low Globo H expression in PR tumors and progressively increased Globo H staining intensity and extent in SD and PD tumors. Scale bar, 6 μm. (B) Quantitative analysis of Globo H expression levels using the H-score method in patient tumors classified as PR (n = 9), SD (n = 22), or PD (n = 50). H-scores were calculated by combining staining intensity and the percentage of positive tumor cells, as described in the Methods. Each dot represents an individual patient sample, and bars indicate mean ± SEM. Statistical significance was assessed by one-way ANOVA with post hoc multiple-comparison testing (*P < 0.05, **P < 0.01). (C) Kaplan-Meier analysis of PFS in GBC patients stratified according to low (n = 59) or high (n = 22) Globo H expression. Patients were dichotomized based on the predefined H-score cutoff described in the Methods. PFS was defined as the time from initiation of gemcitabine-based chemotherapy to documented disease progression or death. Statistical significance was determined using the log-rank test. (D) Kaplan-Meier analysis of OS in the same patient cohort stratified by Globo H expression status (low, n = 59; high, n = 22). OS was defined as the time from initiation of chemotherapy to death from any cause. Survival differences between groups were evaluated using the log-rank test.

To investigate the clinical significance of Globo H expression in advanced unresectable GBC, we performed an immunohistochemical analysis of tumor biopsy samples obtained from 81 patients, all of whom received gemcitabine-based chemotherapy. Globo H expression was detected in 47% of patients, and its expression level was significantly higher in patients with progressive disease (PD) compared to those with partial response (PR) (P = 0.03) or stable disease (SD) (P = 0.007) upon treatment with gemcitabine-based chemotherapy (Figure 6A-B). Survival analysis showed that patients with higher Globo H expression (H-score ≥ 80) had a significantly shorter median progression-free survival (PFS) (2.4 months, 95% CI: 0.9-3.7 months) and overall survival (OS) (median: 5.7 months, 95% CI: 4.5-6.9 months) compared to those with lower expression ( PFS 7.4 months, 95% CI: 4.8-10.1 months; P = 0.0001; OS median: 9 months, 95% CI: 6.9-11.1 months; P = 0.0014) (Figure 6C-D). Moreover, a higher Globo H expression level was significantly associated with adverse clinical parameters, including elevated blood urea nitrogen (BUN) (P = 0.03), reduced hemoglobin levels (P = 0.006), increased total bilirubin (P = 0.038), higher CA19-9 (P = 0.010), elevated CEA (P < 0.001), and increased mortality (P = 0.03) (Table S1). These associations underscore the potential of Globo H as a biomarker of poor clinical outcomes. To further assess the prognostic value of Globo H, we conducted univariate Cox regression analyses, revealing that higher Globo H expression was significantly associated with shorter PFS (HR: 2.96, 95% CI: 1.68-5.19, P < 0.001) and OS (HR: 2.42, 95% CI: 1.38-4.26, P = 0.002) (Table 1). Multivariate Cox regression analyses confirmed Globo H was an independent prognostic factor for both PFS (HR: 2.75, 95% CI: 1.55-4.89, P = 0.001) and OS (HR: 2.17, 95% CI: 1.22-3.88, P = 0.009), even after adjusting for other clinical variables (Table 1). These comprehensive analyses highlight the significant role of Globo H expression as an independent predictor of poor prognosis in advanced GBC and its potential involvement in the GR.

## Discussion

Several studies have explored the mechanisms underlying GR in GBC, focusing on various genetic and molecular factors. One of the key mechanisms is the downregulation of human equilibrative nucleoside transporter 1 (hENT1), which is responsible for the cellular uptake of gemcitabine. Reduced expression of hENT1 has been correlated with poor clinical outcomes in patients with GBC treated with gemcitabine-based therapies 41. Moreover, studies have shown that deoxycytidine kinase (dCK) and ribonucleotide reductase subunits M1 and M2 (RRM1 and RRM2) are key regulators of gemcitabine metabolism and sensitivity, with high levels of RRM1 and RRM2 being associated with increased drug resistance 39, 42. In addition, gemcitabine has been shown to upregulate ABCG2 43, which is responsible for drug efflux and chemoresistance 44. Along this line, our research emphasizes the role of GHCer in upregulating ABCG2 via the A2AR/cAMP/PKA signaling pathway as a novel mechanism contributing to GR in GBC. Our study identified Globo H as a key regulator of ABCG2 expression, which has not been previously explored. Furthermore, we demonstrated that targeting Globo H with monoclonal antibodies or Globo H vaccine effectively reduced ABCG2 expression and enhanced sensitivity to gemcitabine in both *in vitro* and *in vivo* studies.

While other studies have predominantly focused on single genetic factors such as hENT1, dCK, or RRM1/2 in GR, our findings suggest that a broader glycosphingolipid-based mechanism involving Globo H and its downstream signaling through A2AR/cAMP/PKA plays a critical role in modulating GR in GBC. This provides novel and promising therapeutic targets, as blocking A2AR signaling pathways could overcome GR and provide a more effective treatment option for patients with advanced GBC.

Our previous study identified Globo H expression as an independent poor prognostic factor for iCCA 29. Globo H was detected in 86% of tumor samples obtained from 149 patients with resectable GBC by immunohistochemical staining 30. Multivariate Cox regression analysis revealed Globo H expression was an independent poor prognostic factor for disease-free and overall survival in GBC 30. In the present study, we further demonstrated that in patients with unresectable GBC, higher Globo H expression was associated with a poor response to gemcitabine, consistent with the role of Globo H in mediating chemotherapy resistance.

GBC is distinguished from other biliary tract cancers (BTCs) by its unique molecular profile, which underscores the need for targeted therapeutic approaches. Our study demonstrated that Globo H expression was particularly enriched in GBC compared to that in other BTCs 30 and served as an independent poor prognostic marker for resectable GBC. In this study, high Globo H expression in advanced-stage GBC tumor samples correlated strongly with adverse clinical outcomes, including shorter PFS and OS, establishing Globo H as an independent prognostic factor in advanced GBC, in addition to resectable GBC.

The development of a PDX model from a chemotherapy-resistant patient with metastatic GBC provides crucial insights into tumor biology and response to Globo H-targeted therapies. The model demonstrated that the combination of the Globo H-targeting antibody, mAb VK9, with gemcitabine effectively reduced tumor growth, supporting the translational relevance of targeting Globo H in GBC. In addition, the TAA-induced rat CCA model further revealed the role of Globo H in GR through upregulation of ABCG2. These findings align with those of previous studies 29, which highlighted the tumorigenic role of Globo H in iCCA. Both models emphasized the importance of Globo H as a therapeutic target and demonstrated the efficacy of Globo H-targeted interventions in reducing tumor growth and mitigating drug resistance in biliary tract cancers.

We recognize that the TAA-induced rat model used for the combination therapy experiments primarily represents CCA rather than GBC. This model was selected because it provides an immunocompetent BTC system that allows longitudinal PET imaging to monitor treatment response. However, disease-specific differences between CCA and GBC, including anatomical context, stromal composition, and characteristics of the tumor microenvironment, may limit direct extrapolation of these findings to GBC. Therefore, while the TAA model supports the biological relevance of targeting Globo H across BTC, validation in GBC-focused *in vivo* systems such as orthotropic or immunocompetent GBC models will be an important direction for future studies. Another limitation of this study is that the clinical validation was based on a single patient cohort. Although this cohort was carefully curated with comprehensive clinical data, validation in independent multicenter cohorts will be required to confirm the generalizability of our findings.

Recent clinical studies with 1^st^ and 2^nd^ generation Globo H vaccines OBI822/OBI821 and OBI833/OBI821, respectively, have shown encouraging results in other cancer types, particularly in metastatic breast cancer. These studies reported a correlation between strong humoral immune responses, increased anti-Globo H IgG and IgM levels, and improved PFS and OS in patients 28, 40. The promising outcomes in metastatic breast cancer provide the rationale for the ongoing global phase III randomized trial of OBI822/OBI821 in triple-negative breast cancer 45. Phase I trials of OBI833/OBI821 have established its safety, immunogenicity, and preliminary clinical responses 40, 46, paving the way for its application in BTCs. Our preclinical findings that Globo H-targeted therapy could reverse chemoresistance and clinical improvement in a chemotherapy-resistant GBC patient with compassionate use of OBI833/OBI821 therapy support further clinical development of combining chemotherapy with Globo H-targeted therapy in GBC. Currently, a clinical trial of the combination of OBI833/OBI821 with gemcitabine has been initiated in patients with advanced-stage GBC.

However, the GBC patient treated with OBI833/OBI821-showed a mixed response with complete resolution of one lesion, slight reduction of another lesion, and mild enlargement of the largest mass. These findings indicate biological activity of the Globo H-targeted approach but also suggest that vaccine-induced humoral immunity alone was insufficient to achieve comprehensive tumor control in the setting of advanced disease. Although anti-Globo H IgM antibody titers increased during weekly vaccination, the delayed IgG responses and the subsequent protocol specified change of dosing frequency from weekly to every 2 weeks lengthening may have limited sustained immune pressure on rapidly progressing tumors. These observations imply that vaccination, dosing intensity, and baseline tumor burden are critical determinants of clinical efficacy. In patients with extensive disease burden, treatment strategies providing immediate effector function such as monoclonal antibodies or antibody-drug conjugates may be more appropriate than vaccine-based approaches alone.

Globo H expression exhibits substantial inter-patient heterogeneity in biliary tract cancers and is detected in only approximately 40-50% of advanced gallbladder carcinomas. This indicates that Globo H-targeted immunotherapy is inherently applicable to a molecularly defined subgroup rather than the entire GBC population. In this context, tumor Globo H expression itself represents the primary and biologically necessary biomarker for therapeutic targeting, as this strategy directly engages a tumor-associated glycosphingolipid antigen. While additional tumor-intrinsic or microenvironmental factors may influence treatment outcomes, adequate antigen expression level remains the fundamental determinant of therapeutic eligibility. These considerations highlight the importance of biomarker-guided patient stratification when developing Globo H-directed immunotherapies.

## Conclusions

In conclusion, the findings of this study highlight the upregulation of ABCG2 expression by GHCer via the A2AR/cAMP/PKA signaling pathway, contributing to GR, and provide strong evidence for the potential of Globo H-targeted therapies to reverse GR. Given the poor prognostic impacts of Globo H expression in advanced GBC, especially in cases resistant to conventional chemotherapy, targeting Globo H offers a promising therapeutic strategy.

## Supplementary Material

Supplementary figures and table.

## Figures and Tables

**Table 1:** Univariate and multivariate Cox regression analyses of PFS and OS in patients with advanced gallbladder cancer

Variables	PFS							OS					
	Univariate analysis			Multivariate analysis				Univariate analysis			Multivariate analysis		
	HR	95% CI	P-value	HR	95% CI	P-value		HR	95% CI	P-value	HR	95% CI	P-value
Age: ≥ 50 vs < 50	1.52	0.55-4.20	0.417					1.66	0.40-6.81	0.485			
Sex: Male vs Female	0.91	0.57-1.46	0.696					1.47	0.88-2.47	0.142			
Platelet: > 150/≤ 150	1.31	0.83-2.09	0.251					1.30	0.77-2.21	0.333			
BUN: > 25/≤ 25	0.83	0.44-1.59	0.576					0.89	0.45-1.76	0.733			
Creatinine: ≥ 1.3 vs < 1.3	0.99	0.48-2.10	0.998					1.10	0.53-2.30	0.799			
Hemoglobin: < 12 vs ≥ 12	1.43	0.71-2.89	0.315					2.16	0.86-5.46	0.102			
Albumin: < 3.5 vs ≥ 3.5	1.19	0.75-1.89	0.463					1.26	0.74-2.13	0.390			
AST: > 34 vs ≤ 34	1.35	0.84-2.16	0.221					1.25	0.73-2.13	0.413			
ALT: > 36 vs ≤ 36	0.97	0.61-1.54	0.888					1.13	0.67-1.92	0.642			
Total bilirubin: > 1.2 vs ≤ 1.2	1.05	0.65-1.69	0.854					1.11	0.66-1.89	0.693			
ALP: > 96 vs ≤ 96	1.59	0.98-2.57	0.060	1.39	0.85-2.28	0.191		1.81	1.01-3.24	0.047	1.54	0.84-2.81	0.162
CA19-9: ≥ 37 vs < 37	1.45	0.82-2.58	0.205					1.41	0.74-2.67	0.294			
CEA: > 5 vs ≤ 5	1.18	0.73-1.89	0.497					1.09	0.64-1.86	0.751			
Globo H: High vs Low	2.96	1.68-5.19	<0.001	2.75	1.55-4.89	0.001		2.42	1.38-4.26	0.002	2.17	1.22-3.88	0.009

## Abbreviations

ABC: ATP-binding cassette
ABCG2: ATP-binding cassette sub-family G member 2
ADC: antibody-drug conjugate
ADCC: Antibody-dependent cellular cytotoxicity
A2AR: Adenosine A2A receptor
CCA: Cholangiocarcinoma
cAMP: Cyclic AMP
FFPE: Formalin-fixed, paraffin-embedded
GBC: Gallbladder cancer
GHCer: Globo H ceramide
GlcCer: Glucosylceramide
GR: Gemcitabine resistance
IHC: Immunohistochemistry
iCCA: intrahepatic CCA
MALDI-TOF: Matrix-assisted laser desorption ionization-time of flight
mAb: Monoclonal antibody
NK: natural killer
OS: Overall survival
PD: Progressive disease
PDX: Patient-derived xenograft
PET: Positron emission tomography
PKA: Protein kinase A
PR: Partial response
PFS: Progression-free survival
qPCR: Quantitative polymerase chain reaction
Rh123: Rhodamine 123
SD: Stable disease
SUVmax: Maximum standardized uptake value
TAA: Thioacetamide
