# Supplementary material for: Globo H ceramide confers chemoresistance and poor prognosis to advanced gallbladder cancer via A2AR/cAMP/PKA pathway
Source: Theranostics. 2026 Feb 26;16(9):4940–58. doi: 10.7150/thno.126092 (PMC12964375; doi:10.7150/thno.126092)
Supplement: Supplementary file 1 — Supplementary figures and table. [file thnov16p4940s1.pdf]

Supplementary Table 1. Correlation between Globo H expression and clinical parameters in advanced GB patents

Characteristics	Median (range) or (%)	Globo H Expression		*P* value
		Low (n = 59)	High (n = 22)	
Sex				0.905
Male	34 (42)	25 (42.4)	9 (40.9)	
Female	47 (58)	34 (57.6)	13 (59.1)	
Age(years)	66 (37–87)	65 (37–87)	67.5 (43–87)	0.300
Platelet (10^9/L)	164 (17–464)	149 (17–462)	178 (46–464)	0.120
BUN (mg/dL)	12.8 (4–143.8)	11 (4–49.3)	17.2 (7.7–143.8)	0.030
>25	13 (16.0)	10 (16.9)	3 (13.6)	>0.999
Creatinine (mg/dL)	0.68 (0.18–19.3)	0.66 (0.3–10.5)	0.75 (0.18–19.3)	0.130
Hemoglobin (g/dL)	9.5 (1.0–14.1)	9.7 (1.0-14.1)	8.7 (6.6–11.4)	0.006
<12	69 (85.2)	47 (79.7)	22 (100.0)	**0.030**
Albumin (g/dL)	3.5 (0.3-7.8)	3.6 (0.3–4.7)	3.4 (2.5–7.8)	0.154
AST (U/L)	39 (13–2264)	37 (13–1459)	44.5 (19–2264)	0.145
ALT (U/L)	30 (6–1125)	27 (6–1125)	41.5 (6–516)	0.210
Total bilirubin (mg/dL)	0.8 (0.2–32.7)	0.8 (0.3-32.7)	1.2 (0.2–28.2)	0.038
>1.2	30 (37.0)	20 (33.9)	10 (45.5)	0.338
ALK-P	124 (15–1087)	115 (33–1087)	161 (15-975)	0.216
CA19-9 (U/ml)	216 (2–50000)	120.8 (2–22083)	544.5 (20-50000)	0.010
≧37	63 (77.8)	43 (72.9)	20 (90.9)	0.132
CEA, (ng/ml)	7.56 (0.8–2901)	4.9 (0.8–891)	23.2 (1–2901)	<0.001
>5	48 (59.3)	29 (49.2)	19 (86.4)	**0.002***
PFS (months),median (95% CI)	3.4 (2.2–5.2)	5.1 (3.9–6.3)	2.8 (1.6–3.9)	<0.001
No	7 (8.6)	7 (11.9)	0	0.181
Yes	74 (91.4)	52 (88.1)	22 (100.0)	
OS (months), median (95% CI)	7.7 (6.2–9.2)	9.0 (6.9–11.1)	5.0 (4.1–5.9)	0.001
No	22 (27.2)	20 (33.9)	2 (9.1)	**0.026**
Yes	59 (72.8)	39 (66.1)	20 (90.9)	

* Significant difference in multivariate analysis. (odds ratio:4.49, 95% CI:1.28–15.75, *p*=0.013)

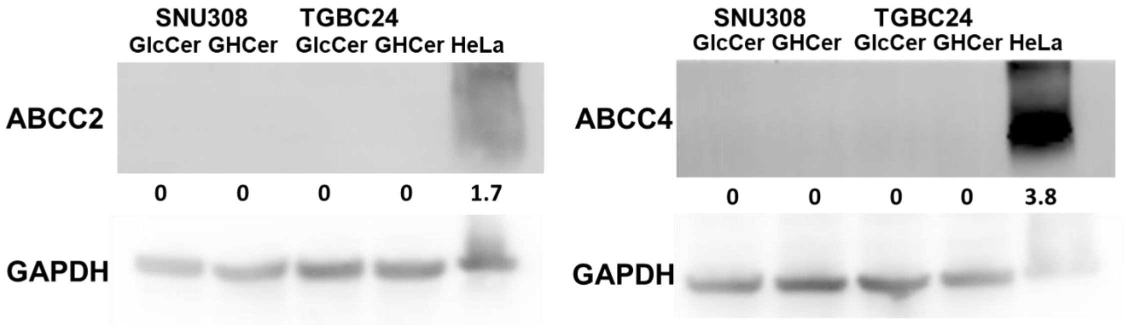

Supplementary Figure 1. GHCer treatment does not significantly alter ABCC2 and ABCC4 expression in SNU308 and TGBC24 cells. Western blot analysis showing the expression levels of ABCC2 and ABCC4 in SNU308 and TGBC24 cells treated with Globo H ceramide (GHCer, 30 μM) or glucosylceramide (GlcCer, 30 μM) for 72 h. ABCC2 and ABCC4 protein expression was minimal in both cell lines and did not show substantial changes following GHCer treatment. Band intensities were quantified by densitometric analysis and normalized to GAPDH. Commercially available HeLa cell lysates (Santa Cruz Biotechnology, sc-2200) were included as positive controls for ABCC2 and ABCC4. GAPDH was used as a loading control.

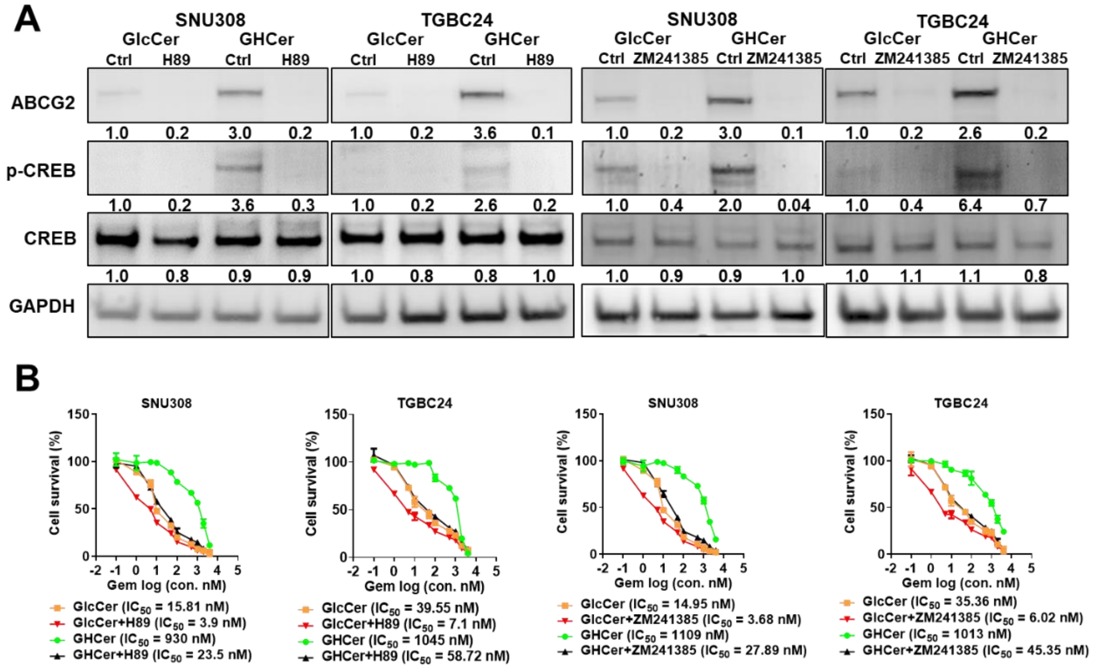

Supplementary Figure 2. Globo H Ceramide Induces ABCG2 Expression and Gemcitabine Resistance through A2AR/PKA Pathway. (A) Western blot analysis of ABCG2, phosphorylated CREB (p-CREB), and total CREB expression in SNU308 and TGBC24 gallbladder cancer cells. Cells were treated with glucosylceramide (GlcCer, vehicle control) or Globo H ceramide (GHCer, 30 μM) for 72 h. Where indicated, cells were co-treated with the PKA inhibitor H89 (10 nM) or the A2A adenosine receptor antagonist ZM241385 (2 μM) during GHCer exposure. Following treatment, whole-cell lysates were prepared and subjected to SDS–PAGE and immunoblotting. GAPDH was used as a loading control. Band intensities were quantified by densitometric analysis and normalized to GAPDH, with values expressed relative to the GlcCer-treated control. (B) Gemcitabine sensitivity was evaluated by dose–response cell viability assays in SNU308 and TGBC24 cells under the indicated treatment conditions. Cells were pretreated with GlcCer or GHCer, with or without H89 or ZM241385, followed by exposure to increasing concentrations of gemcitabine for 72 h. Cell viability was assessed using the alamarBlue assay, and dose–response curves were generated to calculate the half-maximal inhibitory concentration (IC_{50}) values. Data demonstrate that pharmacological inhibition of A2AR or PKA signaling attenuates GHCer-induced gemcitabine resistance.

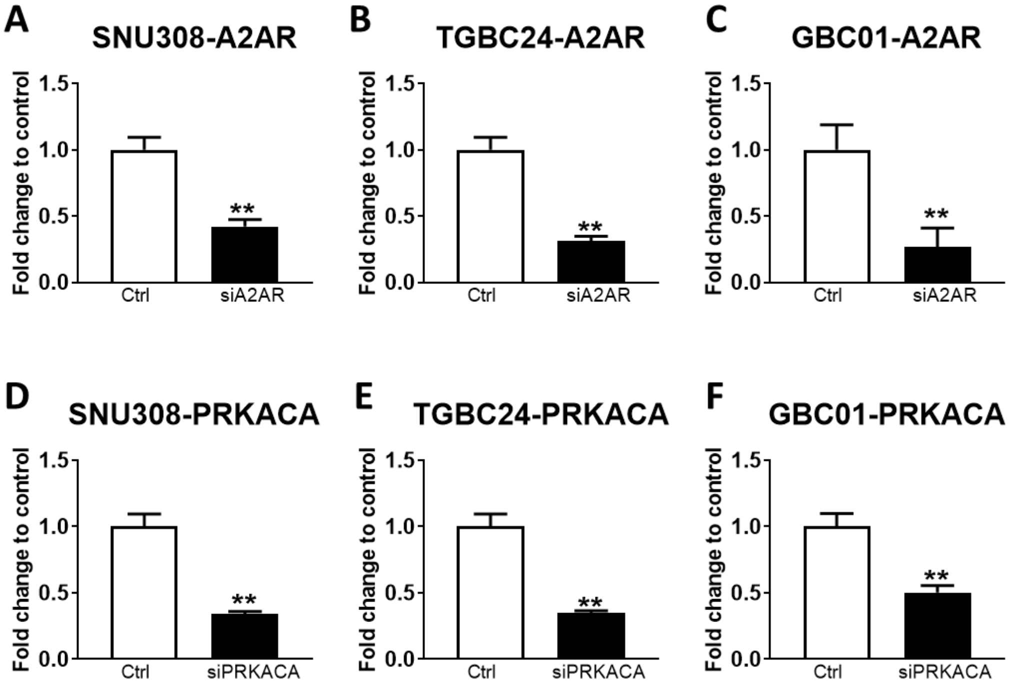

Supplementary Figure 3. Knockdown of A2AR or PKA reduces their expression in gallbladder cancer cell lines.

(A-C) Fold changes in A2AR expression were assessed in SNU308, TGBC24, and GBC01 cells transfected with siA2AR or control siRNA. (D-F) Fold changes in PRKACA expression were evaluated in the same cell lines transfected with siPRKACA or control siRNA. RNA was harvested 72 hours after transfection, and gene expression levels were analyzed by quantitative PCR (qPCR). Data are presented as mean ± SD. $p < 0.01$ compared to control.

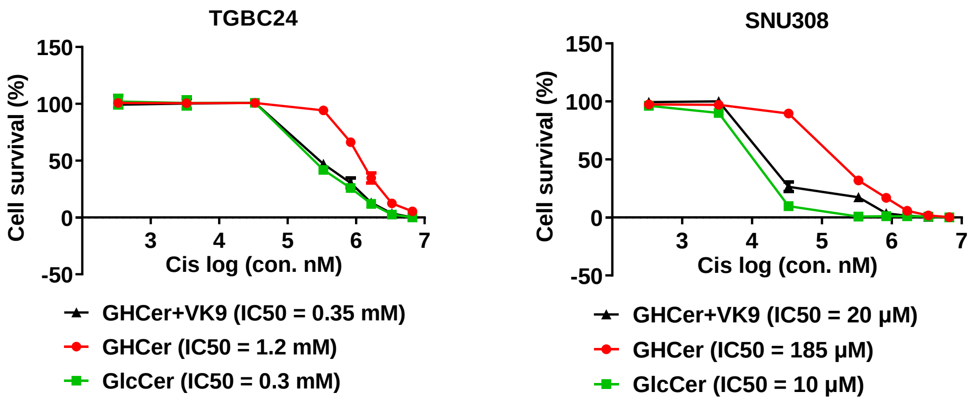

Supplementary Figure 4. GHCer treatment increases cisplatin resistance in gallbladder cancer cells. Dose–response curves showing the effects of different concentrations of cisplatin in the presence of Globo H ceramide (GHCer, 30 μM), glucosylceramide (GlcCer, 30 μM), or GHCer combined with the anti-Globo H antibody VK9 (10 μg/mL) on the survival of TGBC24 and SNU308 gallbladder cancer cells. The half-maximal inhibitory concentration (IC_{50}) values for each condition are indicated. GHCer pretreatment increased cisplatin resistance in both cell lines, whereas VK9 partially restored cisplatin sensitivity.

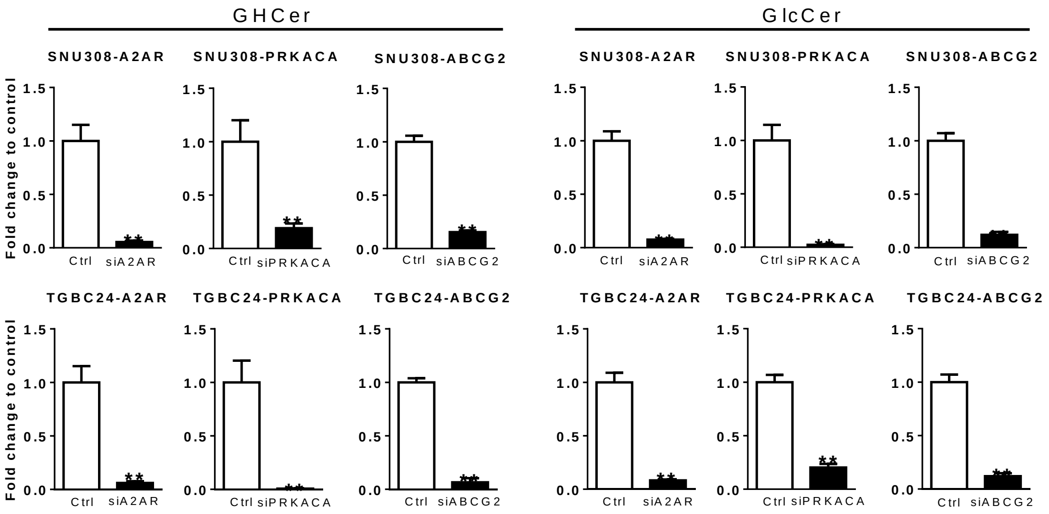

Supplementary Figure 5. Validation of siRNA-mediated knockdown efficiency of A2AR, PRKACA, and ABCG2 used in Figure 2F. Validation of siRNA knockdown efficiency by quantitative RT-PCR (qRT-PCR). Total RNA was isolated from SNU308 and TGBC24 cells transfected with siA2AR, siPRKACA, siABCG2, or siCtrl under the indicated treatment conditions (GHCer or GlcCer). mRNA expression levels of A2AR, PRKACA, and ABCG2 were quantified by qRT-PCR and normalized to GAPDH as an internal control. Data are presented as fold change relative to the corresponding siCtrl group (set to 1.0). Data represent mean ± SEM from at least three independent experiments. Statistical significance was determined using Student’s t-test; P < 0.01.

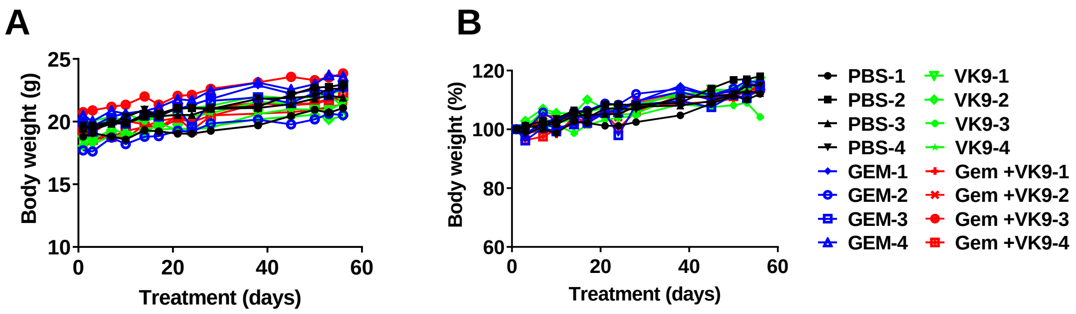

Supplementary Figure 6. Body weight monitoring of NSG mice during treatment. Mice bearing GBC PDX were treated with PBS, gemcitabine (Gem, 10 mpk), mAb VK9 (10 mpk), or the combination weekly for eight weeks (n = 4/group). (A) Absolute body weight was measured weekly. (B) Relative body weight was calculated by normalizing to the initial body weight at day 1.

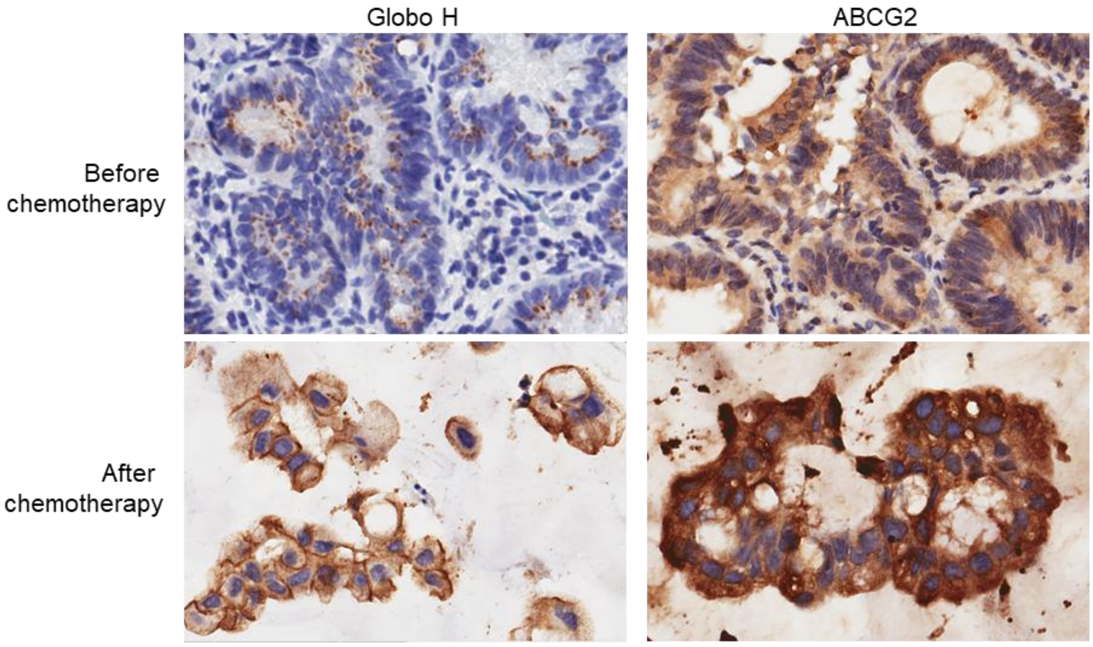

Supplementary Figure 7. Expression of Globo H and ABCG2 in GBC patient tissue before and after chemotherapy, prior to OBI833 treatment. Immunohistochemical staining of Globo H and ABCG2 in GBC patient tissue, comparing expression levels before and after chemotherapy. The top panels show weak expression of Globo H and ABCG2 before chemotherapy, while the bottom panels demonstrate increased expression of both markers following chemotherapy. These findings suggest a possible upregulation of Globo H and ABCG2 in response to chemotherapy, potentially contributing to chemoresistance in GBC.
